# Quantum state revival via coherent energy redistribution

**DOI:** 10.1126/sciadv.ady8981

**Published:** 2026-01-30

**Authors:** Benjamin Crockett, Nicola Montaut, James van Howe, Piotr Roztocki, Yang Liu, Robin Helsten, Wei Zhao, Roberto Morandotti, José Azaña

**Affiliations:** ^1^ Institut National de la Recherche Scientifique – Énergie Matériaux et Télécommunications INRS-EMT, 1650 Lionel-Boulet Blvd., Varennes, QC J3X 1S2, Canada.; ^2^ Department of Electrical and Computer Engineering, The University of British Columbia, Vancouver, British Columbia V6T1Z4, Canada.; ^3^ Augustana College, Department of Physics and Astronomy, Rock Island, IL, USA.; ^4^ Ki3 Photonics Technologies Inc., Montréal, QC, Canada.; ^5^ State Key Laboratory of Ultrafast Optical Science and Technology, Xi’an Institute of Optics and Precision Mechanics, Chinese Academy of Sciences, Xi’an 710119, China.; ^6^ University of Chinese Academy of Sciences, Beijing 100049, China.

## Abstract

Processing and detecting quantum states with high fidelity are essential for enabling quantum advantages across many applications. However, these states are known to be fragile because of their sensitivity to losses, their inability to be amplified, and their susceptibility to decoherence. This makes them far more vulnerable to noise than classical signals, limiting their out-of-lab deployment. We demonstrate quantum coherent energy redistribution, which not only recovers entangled states buried in noise but also improves their properties, moving toward noise-robust architectures with better deployability. Using standard telecommunications infrastructure, we show an order of magnitude increase in the coincidence-to-accidental ratio for time-bin entangled photon pairs. Furthermore, we demonstrate the revival of lost entanglement by recovering quantum interference visibility and fidelity from quantum state tomography measurements of qubits corrupted by noise.

## Teaser

The quantum properties of photons lost due to noise are recovered by coherently redistributing their correlations.

## INTRODUCTION

Noise has been a key concern for experiments in quantum mechanics since the field’s inception. Early experiments on proving the hypothesis of entanglement through violation of Bell inequalities (*1*) emphasized potential loopholes caused by low-efficiency single-photon detectors and the subtraction of accidental counts (i.e., noise) during data analysis (*2*–*4*). Although many of these loopholes have since been addressed, the development of specifically designed experiments (*5*) and the understanding of background noise subtraction techniques (*6*) remain active areas of research as probing the quantum properties of nature necessitates great care to avoid degrading or even destroying the quantum state. Noise continues to restrict the deployment of quantum technologies in both in- and out-of-lab contexts. While classical signals can be amplified and digitized to preserve integrity, quantum wave functions cannot be replicated exactly (*7*) and their digitization/measurement actively alters their state, introducing unique challenges for signal-to-noise management in quantum systems.

Quantum photonics platforms, ranging from microwave to optical, must balance functionality against noise degradation. While recent advances in quantum networking (*8*) enabled major functionalities like secure communications (*9*, *10*), clock synchronization (*11*), and optical quantum computing (*12*–*14*), most of such demonstrations are still performed under strict conditions to minimize the injection of noise photons, such as using dedicated dark fibers. However, quantum signals ideally need to copropagate with classical data streams (*15*–*18*). An alternative solution to fiber-based networks is free-space channels. Although promising, this platform is affected by sunlight (*19*), in turn restricting operations to mainly overnight periods (*9*). Similar problems arise in fields like computing (*12*), metrology (*20*, *21*), spectroscopy (*22*), microscopy (*23*, *24*), and bio-imaging (*25*).

In general, characterizing a quantum state requires performing multiple measurements on identically prepared quantum states. In the case of low-rate photon signals and/or high noise [i.e., low signal-to-noise ratio (SNR)], impractically long-integration times are required to extract the desired quantum information (hours/days in some cases). However, most practical applications in quantum sensing, networking, and computing need measurements to be performed on much shorter timescales. As quantum signals cannot be amplified, reducing loss and noise in these systems is critical for the advancement of quantum information applications beyond the research laboratory. One common approach for noise mitigation involves the discrimination of unwanted counts from the signal, be it in space (*26*), frequency (*9*), and/or time (*27*), through filtering and/or postselection. However, this requires specific prior information about the distribution of the photons, such as their central frequency, time of arrival, bandwidth, or temporal duration. Furthermore, it is very challenging to optimally denoise quantum states that have highly localized distributions, as this requires optical filters with ultranarrow (e.g., sub-gigahertz) frequency passbands and/or temporal gates with ultrashort (e.g., sub-picosecond) time widths. New methods tailored to improve the robustness of quantum states in information processing architectures have been proposed (*16*, *21*, *28*), e.g., using hyperentanglement (*28*), high-dimensional quantum states (*29*), and topologically protected states (*30*). Nonetheless, it would be desirable to have a means for denoising a wave function regardless of the specific nature of the quantum state (single or entangled photons of any dimension). In addition, denoising should be feasible at any stage of a quantum system (i.e., along the transmission path, within a processing unit, at detection, etc.) while requiring minimal information about the input quantum state or noise.

Here, we present coherent energy redistribution as a framework for recovering the nonclassical properties of deteriorated quantum states. We accomplish this using a quantum Talbot array illuminator (qTAI). Originally developed in the context of spatial optics, a TAI consists of a periodic, quadratically varying spatial phase mask followed by diffractive propagation (*31*), akin to an array of lenses (*32*). Through the theory of space-time duality (*33*), Talbot array illuminators (TAIs) have been demonstrated in the time and frequency domains toward, e.g., invisibility cloaking (*34*, *35*), noise mitigation (*36*, *37*), and time-frequency analysis (*38*, *39*). Although there have been demonstrations of space-time dualities applied to quantum systems (*40*–*43*), their implementation on the joint distribution of entangled photons (*44*) remains largely unexplored. In this work, we show noise mitigation of correlated biphotons by compressing the coherent part of their two-dimensional (2D) temporal distribution into short peaks that follow the envelope of the original waveform. Due to its lower coherence, the background noise does not focus into these peaks, allowing effective discrimination of the quantum information through postselection. This enables recovering the quantum properties of the deteriorated states which otherwise would be lost. Here, we demonstrate quantum energy redistribution on the temporal representation of optical waves in the near-infrared region, specifically, the telecommunication C-band. However, the principle can also be readily used in the frequency, spatial, or angular momentum representation of waves (*36*, *45*) through manipulations that can be readily found across the electromagnetic spectrum such as microwaves, visible light, or other physical domains like acoustic and plasmonic systems (*46*).

## RESULTS

### Quantum TAI concept

**Fig. 1.**
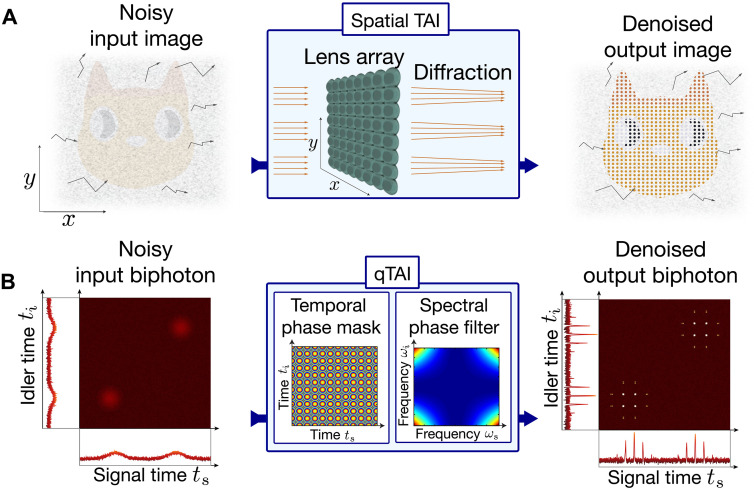
Quantum wave function denoising via the concept of coherent energy redistribution. (**A**) A TAI, analogous to a lens array, redistributes a spatial image into an array of spots. This device focuses coherent light rays, represented by orange arrows in the image of interest, that are approximately parallel to each other within the extent of a lens. In contrast, the incoherent noise components (gray) point in random directions and are not focused, allowing to discriminate the image of interest from the noise background. (**B**) Recovery of a deteriorated quantum state using the qTAI, here shown in the temporal domain. The joint distribution of the biphotons undergoes two successive (2D) phase manipulations, namely, temporal phase modulation and dispersive propagation, respectively equivalent to a 2D lens array and free-space diffraction processes. The temporal phase mask can conveniently be limited to a phase excursion of 2π on each photon for practical implementation via electro-optic technologies. The excursion of the spectral phase profile can easily extend over hundreds of π through available dispersive elements. This leads to an energy redistribution in the joint distribution, allowing for denoising via postselection on the output peaks.

The coherent energy redistribution mechanism that enables noise mitigation in the qTAI is related to the working principle of a focusing lens array. Consider a 2D wavefront imprinted with an image (here, a cat) as shown in Fig. 1A. The relative k vectors (i.e., the tilt of the orange rays coming from this image) are considered to be nearly parallel to each other within the extent of each of the individual lenses in the considered array. Upon propagation through this array of thin lenses, the rays coherently acquire a quadratic phase variation such that rays near the edges of each lens will become more skewed toward the center. Subsequent diffractive (free-space) propagation will lead to the focusing of these rays into narrow spots at a distance corresponding to the focal point of the lenses. The resulting waveform preserves the profile of the original image if the lenses are sufficiently close to each other. Specifically, adjacent lenses must be spaced by a distance lower than the smallest features of the original image, according to the well-known Nyquist criterion (*37*, *47*). Each thin lens in the array introduces a continuously increasing quadratic phase variation along the incoming wavefront, whereas a TAI uses a discrete and bounded phase shift following a Talbot phase profile to achieve a more efficient focusing performance (*48*), as further described below.

If we now consider that the input image is corrupted by incoherent noise, with k vectors pointing in random directions (gray arrows), then the latter will not acquire the deterministic quadratic wavefront induced by the lenses and will not focus. Instead, such incoherent noise remains evenly distributed at the output plane. This, in turn, leads to a local increase in contrast between the coherent image and the incoherent noise background, effectively increasing the local (i.e., in the spots) SNR of the output wavefront.

The same principle can be readily applied to manipulate the joint distribution that characterizes a given correlated quantum state (*49*, *50*), by implementing phase transformations along the corresponding domains or degrees of freedom (time, frequency, space, etc.) (*45*); see Fig. 1B. This results in a local contrast increase between signal and background noise while preserving the original signal profile. We consider, without loss of generality, the case of time-bin entangled photons. The scheme relies on a phase modulator (PM), implementing Talbot phase modulation on incoming photons along the time domain, followed by a group-velocity dispersive medium [here, a linearly chirped fiber Bragg grating (LCFBG)], realizing a quadratic phase manipulation along the frequency domain. Photons from all wavelength channels entering the qTAI are processed simultaneously and are affected by nearly identical phase transformations, ensuring that the desired coherent energy redistribution is applied along the photons’ joint correlation functions.

### Experimental implementation

Fundamentally, the phases used in a TAI can be derived from number theory (*37*, *51*, *52*). The distinctive feature is that the phase modulation profiles can effectively be bounded to a 2π maximum phase excursion for a more efficient focusing approach when compared to conventional lens systems (*44*, *48*). The most general implementation of a TAI consists of a sequence of two discrete phase manipulations, along the temporal and spectral (frequency) domains, respectively, where one of these can be made continuous for convenience, still achieving a similar effect. In the work reported here, we implement the temporal qTAI using a discrete temporal phase modulation, followed by a continuous spectral phase manipulation. The qTAI could also be implemented using a continuous phase manipulation followed by a discrete one, which is generally more convenient to process waveforms in the frequency domain (*36*).

We describe here how to use the qTAI to redistribute a temporal wave function into peaks of width $t_{p}$ separated by $t_{q}=qt_{p}$, where q is the compression or enhancement factor of the denoising scheme, proportional to the targeted amount of denoising. This implements a filtering scheme with an effective noise mitigation bandwidth of $1/t_{q}$ (see discussion below on the frequency-to-time mapping effect). We note the term “effective noise mitigation bandwidth” because the TAI cannot be described as a linearly time-invariant system, as it is the case for conventional frequency filters (*47*). First, the temporal phase of the signal is modulated following a periodic pattern of length $t_{q},$ composed of q bins of width $t_{p}$ satisfying $\varphi_{n}=n^{2}\pi \left(q-1\right)/q$, where n=1,…,q. This is then followed by linear dispersive propagation, where the slope of the group delay versus angular frequency given by the second-order dispersion coefficient $\ddot{\phi }$ satisfies $\ddot{\phi }2\pi =qt_{p}^{2}$. Similarly to a time-lens array scheme, this procedure enables focusing the signal energy extending over each phase modulation period $t_{q}$ into short pulses of width $t_{p}$, such that, ideally, each output pulse exhibits a peak power about q times larger than that of the input signal at the corresponding time location. A similar focusing effect is accomplished on the joint temporal distribution that characterizes an entangled biphoton state. In this case, the temporal phase manipulation can be modeled as a 2D phase as $\text{exp}[i\;\left(\varphi_{n_{s}}+\varphi_{n_{i}})\right],$ where the indices $n_{s}$ and $n_{i}$ are orthogonal to each other, as shown in Fig. 1B. The dispersive propagation can similarly be modeled as a 2D spectral phase mask (*53*), which leads to the formation of a 2D array of peaks that samples the input joint temporal distribution. For a derivation of the energy redistribution process implemented by the qTAI on the joint temporal distribution of a biphoton state, we refer the readers to Supplementary Text 1.

**Fig. 2.**
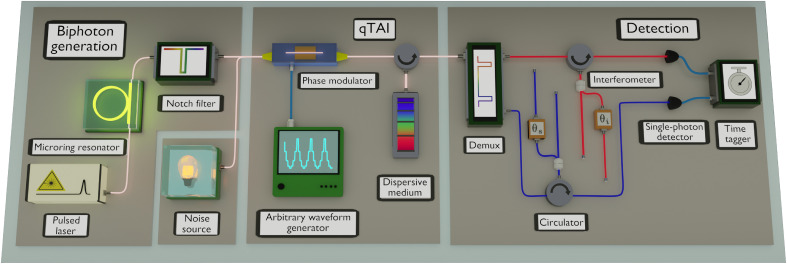
Experimental setup. Denoising biphotons generated by spontaneous four-wave mixing (SFWM) in a silicon nitride microring resonator (MRR) pumped by a pulsed laser. To characterize quantum interference between entangled photons, we use a sequential time-bin scheme where the interferometer provides a delay equal to the laser repetition rate. Piezo-actuators are used to scan the relative phases $\theta_{s}$ and $\theta_{i}$ between the two arms for the signal and idler photons, respectively.

**Fig. 3.**
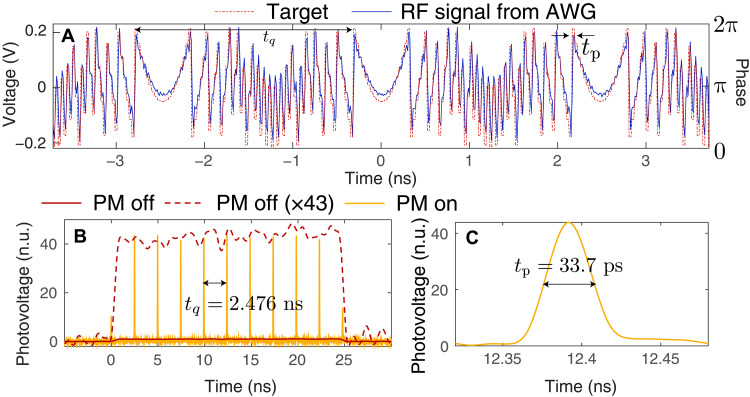
Characterization of the temporal TAI performance on classical signals. (**A**) Radio frequency (RF) signal generated by the AWG used to drive the electro-optic PM for the qTAI, as measured by a 28-GHz real-time oscilloscope (blue trace, left *y* axis), compared to the theoretically designed phase pattern (dashed red trace, right *y* axis). (**B**) Classical characterization of the energy redistribution of a ~25-ns square pulse, with a measured amplification factor of 43, as shown by the scaled version of the waveform measured at the output of the sampling module with the PM off. The vertical scale is shown as normalized units (n.u.). The TAI pulses are separated by $t_{q}=2.476\;n$s, featuring a pulse intensity full width at half maximum (FWHM) of 33.7 ps, as shown in (**C**).

As depicted in Fig. 2, we implement the necessary temporal phase manipulations using an electro-optic PM driven by an electronic arbitrary waveform generator (AWG), providing TAI phases with q=77 and $t_{p}=32.2$ ps. The phase modulation signal generated by the AWG is shown in Fig. 3A, compared to the ideal phase profile. This manipulation is followed by an LCFBG inducing a second-order dispersion of $\ddot{\phi }\approx 12,758$ ps^2^.

A classical characterization of the implemented TAI is shown in Fig. 3 (B and C), optimized such as to maximize the energy within each peak. This results in a compression factor of 43, measured as the ratio of the maximum value of the TAI peak with the PM turned on (PM on) to the maximum value of the signal measured at the output of the TAI system with the PM turned off (PM off), effectively neglecting the insertion loss of the device. We believe that this reduction from the designed enhancement factor of q=77 to the experimentally observed factor of 43 is due to the imperfect phase modulation caused by limited temporal bandwidth and higher-order dispersive terms. These nonidealities will, in turn, lead to the formation of satellite peaks (i.e., energy that is not properly focused into the TAI peaks; see fig. S1), translating into a reduction of the correlated counts after denoising.

The specific design reported here is aimed at denoising relatively narrowband waveforms extending over a temporal duration of ~2.48 ns or longer (given by $t_{q}$), such as those generated by resonant structures [e.g., microring resonators (MRRs) (*54*, *55*)] or cold atomic gases (*56*). We note that, as the waveform extends over substantially longer durations than $t_{q}$ (e.g., ~4 times longer than $t_{q}$ or more), the TAI peaks are shaped more accurately, displaying the theoretically expected shape, width, and peak value, closely outlining the input waveform profile with the predicted relative intensity increase (*57*). The energy redistribution from the qTAI can be considered as a form of lossless wave sampling, and, as such, the separation of the output peaks must satisfy the Nyquist sampling criterion to the fastest variations of the input wave. In particular, the bandwidth of the input waveform should have a bandwidth less than $1/t_{q}$ on timescales $\sim t_{q}$ for the peaks properly perform the sampling process.

Because the noise has variations that do not meet the Nyquist criterion, it does not undergo the same focusing effect. Therefore, the qTAI enables a highly efficient discrimination of the slowly varying coherent wave components from incoherent temporal variations changing faster than $\sim 1/t_{q}$. The specific scheme reported in our work allows effective discrimination of temporal noise variations down to the MHz range (i.e., 1/2.48 ns = 403.3 MHz), which is deteriorated to ~1.16 GHz due to the limited temporal resolution of the superconducting nanowire single-photon detectors (SNSPDs; see discussion below). In terms of the joint distribution, to preserve the general mode structure of the quantum state, the sampling criterion also needs to hold in the higher-dimensional space of the biphoton state (see discussion on the Schmidt number in Supplementary Text 2). In particular, the qTAI generates a 2D array of spots, here in the temporal representation of the joint distribution, as shown in Fig. 1B. To preserve the mode dimensionality of the quantum state, the joint distribution also needs to be fully captured by the qTAI samples in this 2D space (i.e., satisfy the Nyquist criterion in 2D). We emphasize that the 1D phase transformations are applied to signal and idler simultaneously, thus implementing a 2D transformation in the joint distribution across different wavelength channels.

Hence, the noise mitigation performed by the qTAI can be described through a time-frequency approach, depending on the relative bandwidths of the quantum state and the background noise. It has recently been shown that the TAI framework allows for time-frequency analysis (*38*, *39*), alongside the noise mitigation capabilities shown here. In particular, it enables continuous mapping of the spectrum of an optical signal onto the time domain over each time period $t_{q}$ following the well-known frequency-to-time mapping law $\nu_{t}=\bar{t}/\left(2\pi \ddot{\phi }\right)$, where the time $\bar{t}$ is taken relative to the center of each window of width $t_{q}$. Thus, because the wave function to be denoised covers a bandwidth of $1/t_{q}$ or less, it is concentrated into a short pulse of width $t_{p}$ at the center of each phase modulation window.

In other words, because the Nyquist criterion indicates that the signal to be denoised remains approximately constant throughout the phase modulation window $t_{q},$ the spectrum of the signal is mapped onto the time domain as the dc (or 0-frequency) component of each frequency-to-time mapped spectrum. On the other hand, the broader noise will be mapped to a much larger temporal duration. If the noise bandwidth $\Delta \nu_{n}$ is much larger than the modulation bandwidth $1/t_{p}$, then it will be distributed evenly throughout multiple phase modulation windows of duration $t_{q}$. If, on the other hand, $\Delta \nu_{n}<1/t_{p}$, then the noise will be mapped onto the time domain with a duration $<t_{q}$ following the frequency-to-time mapping law $\Delta t_{n}=2\pi \ddot{\phi }\Delta \nu_{n}$ (*58*). This translates into a reduction of the achievable enhancement factor to ~$\Delta t_{n}/t_{p}$ instead of $t_{q}/t_{p}$. Still, the noise mitigation bandwidth remains as $1/t_{q}$ because the counts are postselected within each peak of duration $t_{p}$, corresponding to a frequency-to-time mapped bandwidth $t_{p}/\left(2\pi \ddot{\phi }\right)=1/t_{q}$ from the definition of the second-order dispersion coefficient given above.

Therefore, to establish a more effective filtering scheme, the noise mitigation bandwidth of the qTAI, given by 1/$t_{q}$, should approach the frequency bandwidth of the signal to be denoised. This represents a trade-off between the specifications of the qTAI scheme for optimal filtering and the fidelity of the resulting output peaks that outline the profile of the input waveform. When implementing the qTAI system, the bandwidth of the quantum state should be known for optimal denoising. However, knowledge of the central frequency is not necessary because all wavelengths are processed simultaneously, as long as it is within the operation bandwidth of the qTAI (i.e., electro-optic PM and dispersive line, in the implementation considered here). Furthermore, no specific knowledge on the time-of-arrival or temporal duration of the quantum state is required, and there is no limitation on the dimensionality of the quantum state (qubit, qudits, etc.). The central frequency of the noise does not need to be known, but the noise bandwidth should be larger than that of the quantum state if both share the same central frequency. This implies that noise contributions that have the same time-frequency distribution as the signal of interest (such as multiphoton events generated from the same pump pulse and in the same resonance as the biphoton) undergo an identical focusing process and cannot be mitigated using this method.

### Experimental results

Proof-of-concept demonstrations of quantum energy redistribution are carried out on infrared biphotons (~1550 nm) using off-the-shelf telecommunication components. As our source, we leverage a well-established biphoton generation process in which time-bin entangled states are produced at different optical wavelengths via spontaneous four-wave mixing (SFWM) in integrated MRRs (*54*, *55*) (see Materials and Methods and fig. S2 for details about the experimental setup). We show substantial improvement of several key metrics, including the coincidence-to-accidental ratio (CAR), Schmidt number, quantum interference visibility, and fidelity. While our focus is on improving the detection of noisy biphoton states, the technique can be extended to any stage of a quantum information architecture, such as for quantum state generation or transmission instead of detection (see Materials and Methods). While all measurements reported in what follows are performed with synchronization between the source and the qTAI, we demonstrate that this is not necessary for enhancing quantum performance metrics, as supported by the CAR improvements shown in Supplementary Text 3 and fig. S5.

Although we focus here on mitigating optical noise, detector-generated dark counts can be an important source of noise for certain applications, particularly those relying on avalanche photodetectors. These detectors operate at room temperature but are associated with much higher dark counts than SNSPDs. This type of noise is typically mitigated by temporal gating, while optical or electrical frequency filtering methods do not represent a potential solution to this problem. As detailed in fig. S6, by compressing the signal of interest into narrow time bins, the qTAI is able to shrink the window of observation and thereby reduce the dark count noise through temporal gating.

We assess the denoising effect by first measuring the CAR under different pumping conditions (quantified by the resulting mean photon number) and noise injection rates, with the interferometers removed from the setup in Fig. 2 (see Materials and Methods for details on the data analysis). The injected noise rate is controlled using a variable optical attenuator (VOA) on the incoherent amplified spontaneous emission noise provided by an erbium-doped fiber amplifier (EDFA), filtered to two 9-GHz bands centered at the signal and idler frequencies, respectively. This emulates an external source of stochastic thermal noise, such as sunlight in satellite communications (*9*) or broadband Raman noise in optical fibers (*59*). The 9-GHz filtering reduces the input waveform noise to about the lowest level that can be achieved using standard optical band-pass filtering methods (*60*). The resulting signal is then sent to the qTAI, with the specifications defined above, which processes all channels simultaneously. The processed resonances are then demultiplexed by a multiport programmable filter (Demux) and directed to SNSPDs connected to a time-correlated single-photon counter.

**Fig. 4.**
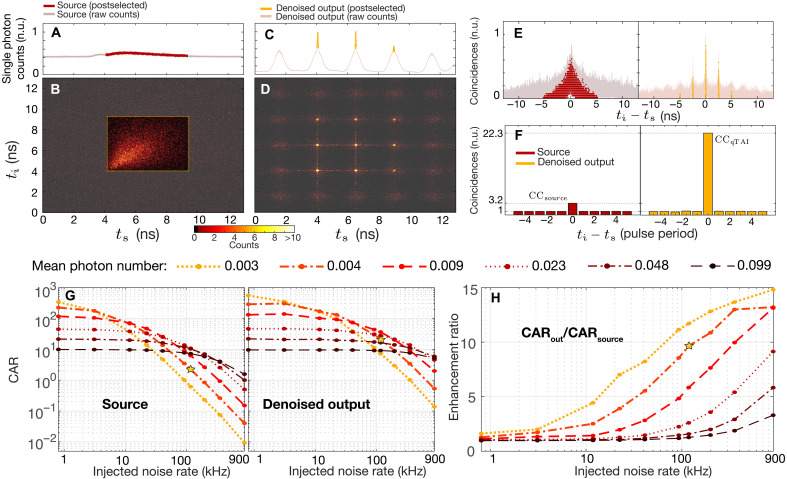
Denoising of biphotons and CAR improvement. (**A**) Histogram of the single counts for the input signal photon. The idler photon follows a similar distribution. (**B**) Associated biphoton temporal distribution, with the postselection window outlined in yellow. (**C** and **D**) same as (A) and (B), but at the output of the denoising module, leading to the expected formation of the TAI peaks. (**E**) Effect of the TAI peak formation on the histograms of the coincidences versus the delay between the photons. (**F**) Histogram of postselected correlated counts versus pulse separation, normalized to their respective accidental counts, displaying a significant improvement in the CAR. (**G**) CAR of the input and denoised output, as a function of injected noise rate, shown for various mean photon numbers. (**H**) Enhancement of the CAR, expressed as a ratio of the output CAR over the input CAR. The results shown in (A) to (F) correspond to the setting depicted by the yellow star.

We first demonstrate the energy redistribution effect on a moderately noisy waveform in Fig. 4 (A to F), where the mean photon number of the source is set to 0.004 and the injected noise count rate is ~118 kHz (see Materials and Methods). The single-photon histogram of the signal photon measured at the source after noise injection (i.e., before the qTAI system in Fig. 2) is shown in Fig. 4A, while the associated biphoton temporal distribution is shown in Fig. 4B. We consider the counts underlined by the red trace, discarding the rest. Sending this state through the denoising system leads to the formation of qTAI peaks on each single-photon histogram, as shown in Fig. 4C for the signal photon (yellow trace). This, in turn, “focuses” the 2D biphoton temporal distribution into localized spots with increased correlated counts relative to the background noise; see Fig. 4D. Because the noise covers a much narrower spectral bandwidth than the qTAI modulation (9 GHz versus 31.1 GHz, respectively), it is redistributed into ~800-ps-wide pulses at the base of the qTAI peaks due to the frequency-to-time mapped effect described above. Along with the deterioration of the temporal resolution, this leads a noise reduction factor of ~8.2. See Supplementary Text 4 for further details, including a mathematical description of the denoising effect on incoherent and coherent (e.g., multiphoton events) noise contributions that are also discussed.

The number of coincidences as a function of the difference in arrival times between the idler and signal photons $t_{i}-t_{s}$ measured on the source (i.e., before the qTAI module) is shown in Fig. 4E. After traveling through the denoising module, the energy of the biphoton is redistributed, resulting in noise mitigation (yellow trace). Limiting the analysis to the counts within the TAI peaks yields the curves highlighted in yellow, which allows for a significant amount of noise counts to be discarded. We also present the coincidence counts (CCs) as a function of pulse separation in Fig. 4F, indicating a clear increase when normalized against the accidental counts (ACs). This translates into a net increase in the CAR=(CC−AC)/AC (*61*) from 2.2 to 21.3, comparing before and after the qTAI module, respectively, representing a 9.6-fold improvement.

Measurements of the denoising effect of quantum energy redistribution on the CAR for various mean photon numbers and noise injection rates are shown in Fig. 4G (see sample histograms in Supplementary Text 5, with figs. S9 to S11). The noise-induced accidental counts are significantly reduced by the qTAI, allowing the observed increase in CAR (shown in fig. S12). Moreover, note that a single qTAI module can process multiple resonances in a self-tracking fashion. This circumvents the need for any form of spectral alignment, which can be difficult to implement with narrowband filtering approaches. We leverage this capability to recover the spectral correlations of entangled states, thus improving their Schmidt number, as shown in Supplementary Text 2.

**Fig. 5.**
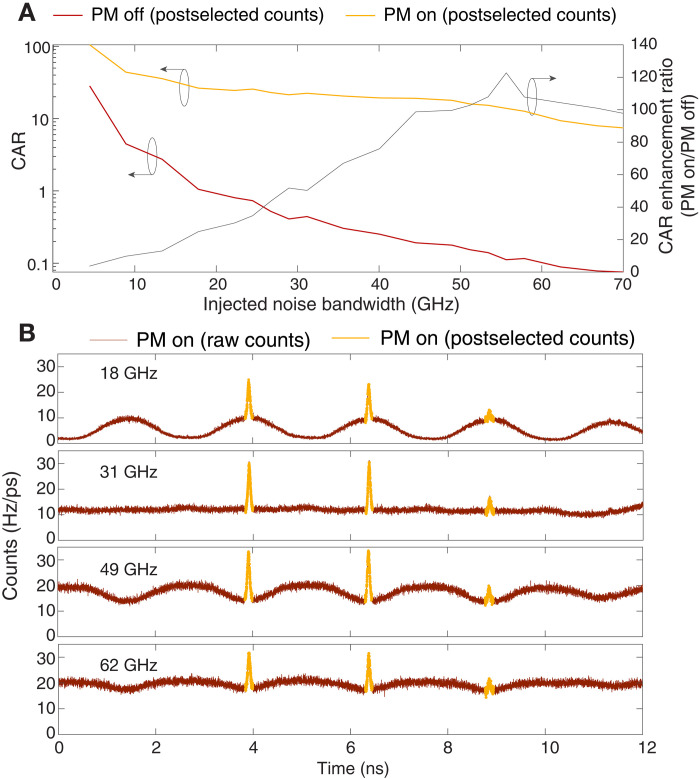
CAR enhancement versus injected noise bandwidth. The VOA is configured to produce an injected noise rate of 92.8 kHz at 9 GHz, and the mean photon number is set to $\bar{n}=0.009$. (**A**) Trend of the CAR for PM off (red trace, left axis) and PM on (yellow trace left axis), and ratio of PM on/PM off (black trace, right axis). (**B**) Example histogram traces for PM on with increasing noise bandwidths, indicated by the legend on each graph. A similar histogram with a noise bandwidth of 9 GHz is shown in Fig. 4C. Notice how the shape, width, location, and height of the background noise change with varying noise bandwidth.

Figure 5 shows how the CAR enhancement varies with the noise bandwidth, comparing the output of the qTAI system with the phase modulation turned on and off. The enhancement increases significantly as the noise bandwidth rises and then plateaus. Histograms of the signal photon with PM on are presented in Fig. 5B, with the noise bandwidth indicated by the legend. In the case of PM off, the histograms simply show an increase of the noise floor with increasing noise bandwidth. On the other hand, in the PM-on case, one can see that the profile of the background noise depends on the noise bandwidth. Narrowband noise is redistributed into peaks where the minima have relatively low count rates that approach that of the detector dark counts, as depicted with a noise bandwidth of 18 GHz in the first panel of Fig. 5B. This noise background becomes relatively uniform at 31 GHz, corresponding to the phase modulation bandwidth, because this noise is redistributed into peaks of duration ~$t_{q}$ per the frequency-to-time mapping law. After this point, neighboring noise distributions start to overlap, giving rise to higher noise between the qTAI peaks, as depicted with a noise bandwidth of 49 GHz, leading to a steeper slope in the enhancement of the CAR versus noise bandwidth. As the noise bandwidth becomes much larger than the modulation bandwidth, the shape of the background noise becomes relatively flat regardless of the bandwidth, as shown with a noise bandwidth of 62 GHz, leading to the observed plateauing of the enhancement versus noise bandwidth. In Supplementary Text 6 and fig. S14, we show an analogous analysis to that shown in Fig. 4 (G and H), but where the noise has a bandwidth of 62 GHz instead of 9 GHz. This allows for even higher noise mitigation factors, demonstrating that the qTAI outperforms a 9-GHz band-pass filter (BPF).

An important feature of the qTAI is its ability to revive the quantum entanglement properties of a biphoton state corrupted by noise. As an entangled state is introduced into a noisy environment, it changes into a separable state, and the entanglement properties are lost (*62*). By setting the interferometric delay larger than the single-photon coherence length such that there is no single-photon interference, but shorter than the two-photon coherence length, coincidence fringes with a minimum visibility of 71% are required toward violating the Clauser-Horne-Shimony-Holt (CHSH) Bell inequality, a commonly used criteria for entanglement verification by eliminating the possibility of local hidden variables (*1*, *63*). Revival of entanglement is demonstrated through an improvement of the visibility observed in quantum interference measurements of a sequential time-bin entangled source (*64*, *65*), using one of the interferometers shown in Fig. 2 of the detection stage, where both signal and idler photons are processed simultaneously (see Materials and Methods).

**Fig. 6.**
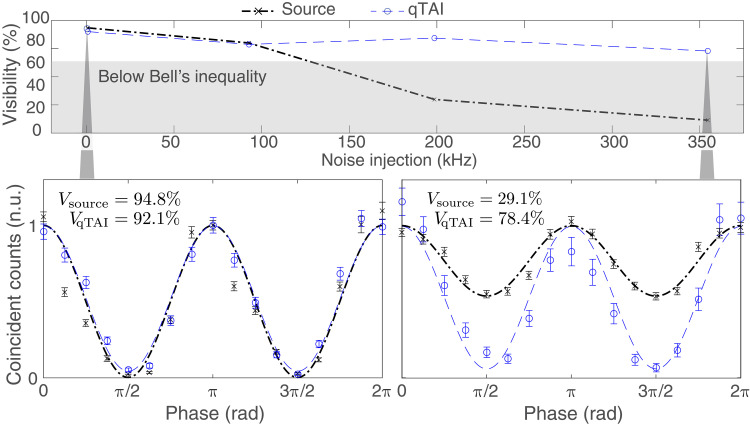
Quantum interference visibility improvement from the qTAI. The visibility of the input quantum state rapidly falls below the Bell inequality threshold with increasing noise injection, while the entanglement persists when processed by the qTAI module, allowing for an increase in visibility by up to 49.3%. The insets show the quantum interference curves for the source and output of the qTAI module at different noise injection rates.

**Fig. 7.**
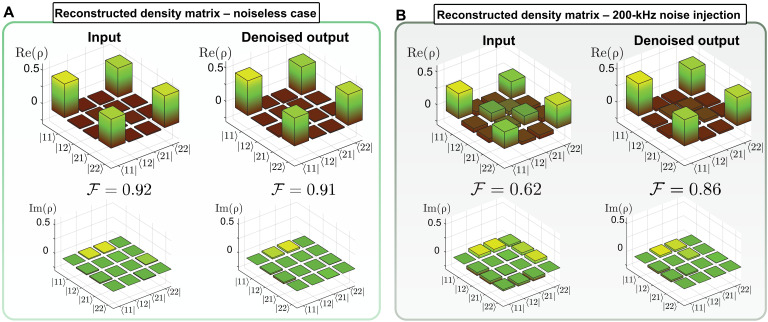
Quantum state revival measured through quantum state tomography. (**A**) Without any noise injection, both the input and the output of the qTAI denoising system show a similar fidelity of >0.9. (**B**) The quantum state is buried under noise, reducing the fidelity of the input (source) to 0.62. The quantum state is recovered with a fidelity of 0.86 using the qTAI.

The results are presented in Fig. 6 for various noise rates (with a bandwidth of 9 GHz) and a mean photon number set to 0.004. The visibility of the source rapidly falls below the minimum threshold of 71%, while entanglement persists after qTAI processing even under significantly higher noise rates (see Materials and Methods). This suggests that the qTAI could be used to establish quantum communication links in noisy environments (see fig. S7 for a proof-of-concept experiment on a simplified quantum key distribution (QKD) link (86). Last, in Fig. 7, we display the recovery of a noisy time-bin entangled state through quantum state tomography (QST) (*66*, *67*). In this case, both interferometers from Fig. 2 are used to process the signal and idler photons individually (see Materials and Methods). The noiseless case shows similar performance at both the input and output, as illustrated in Fig. 7A. However, when 200 kHz of noise with a bandwidth of 9 GHz is introduced, the fidelity decreases to 0.62 for the input but remains high at 0.86 for the output as shown in Fig. 7B, demonstrating the qTAI’s ability to recover a deteriorated quantum state.

## DISCUSSION

Here, the achievable compression factor q is limited by the timing jitter ($t_{\text{jit}}$ ~ 50 ps) of our SNSPDs. Thus, we measure the qTAI peak full width at half maximum (FWHM) to be ~60 ps, resulting in a measured q of ~41, given by the ratio of the peak separation to the peak width. This leads to a deterioration of the noise mitigation process, because the output pulses (of width $t_{p}$) need to be properly resolved for optimal temporal postselection and, hence, denoising. Specifically, if the qTAI peaks are broadened to a duration $t_{\text{jit}}>t_{p}$, then there is a deterioration of the noise mitigation bandwidth, which increases to $t_{\text{jit}}/\left(2\pi \ddot{\phi }\right),$ evaluated to 1.16 GHz in this work rather than the optimal value of $t_{p}/\left(2\pi \ddot{\phi }\right)=1/t_{q}=403.3$ MHz (see Supplementary Text 4 for details). Thus, to optimally exploit the denoising capabilities of the qTAI, the temporal resolution of the single-photon detectors should match be finer than $t_{p}$. Note that this temporal filtering step for the postselection could alternatively be implemented in an analog fashion directly on the quantum state rather than after detection, e.g., by using a temporal gate to suppress the background noise between the peaks (*27*, *68*). This would remove the requirement for the output pulses to be properly resolved by the detection scheme at the cost of higher attenuation from the gating stage. Using such an approach could allow for phase manipulations to be implemented via state-of-the-art electro-optic technologies (*69*) or nonlinear techniques (*70*) for higher compression factors and for denoising photons with ultrafast temporal distributions. A further extension of this technique could also include the possibility of obtaining a continuous denoised wave function rather than the qTAI peaks if a narrowband waveform is needed after denoising. This can be accomplished by applying inverse phase manipulation (i.e., temporal and spectral phase modulations of opposite sign to the implemented qTAI design) after removing the noise between the peaks (*34*, *35*). This approach could be particularly relevant if further processing is needed on the denoised state, for instance, if it needs to interact with other narrowband platforms such as superconducting systems (*71*) or trapped ions (*72*).

The insertion loss from the denoising module used here is estimated to be ~5.3 dB (2.8 dB for the PM and 2.5 dB for the LCFBG). We note that a loss-optimized implementation of these components could reduce losses to around 2 dB, using electro-optic modulators with ~1-dB loss (*73*), commercially available low-loss circulator with <0.4-dB total roundtrip loss (*74*), and fiber Bragg gratings with losses <0.3 dB (*75*). Reducing the insertion losses of the module would allow to approach the performance observed comparing with the measurements at the output with the PM off; see fig. S13. Alternatively, considering propagation through a deployed optical fiber network, the dispersion naturally provided by the fiber could be harnessed to avoid the use of dedicated dispersive elements (e.g., LCFBGs or dispersion compensating modules) and allow for even lower insertion loss values when implementing the qTAI.

In closing, we have introduced coherent energy redistribution on quantum wave functions, enabling noise mitigation in these systems. We have used the qTAI for joint distribution measurements of correlated photons, showing an unprecedented increase in the measured CAR values by over 10 times with respect to a noisy input quantum state. We have also demonstrated that entanglement recovery from the qTAI enhances the quantum interference visibility by up to 49.3% and leads to an increase in fidelity of 0.24 for noisy time-bin entangled photons. This technique could enable the practical deployment of quantum systems in real-world noisy environments, as well as increase capacity for foundational research like loophole-free tests or experiments aimed at the study of quantum effects requiring extremely low noise detection. We believe that the proposed framework could accelerate the development of other important functionalities on entangled photon pairs beyond denoising, such as for efficient light-matter interaction (*42*), quantum computing (*76*), or quantum state characterization (*77*).

## MATERIALS AND METHODS

### Experimental details for biphoton generation

To demonstrate the denoising capabilities of the qTAI, we show its use on photonic time-bin entangled states generated from an MRR (*55*). Specifically, an MRR generates photon pairs (signal and idler) in well-defined resonances, here with ~400-MHz line widths and 100-GHz free spectral range, which exhibit strong spectral and temporal correlations due to energy and momentum conservation in the photon pair generation process (*54*). MRR technology has attracted interest in recent years because of the narrow bandwidths of the photons, making them suitable for integration with other quantum infrastructures like trapped atoms (*78*), and because the resonances can be used as telecommunication channels aligned to the standard telecommunication grid (*79*).

A detailed schematic of the biphoton generation setup is shown in fig. S2A, and a summary of the main experimental parameters is presented in Table 1. All fiber-optic components are polarization maintaining to ensure stable operation over extended periods. The pump beam is generated by modulating a continuous-wave laser (NKT Koheras BASIK E15, centered at 1550.1 nm) with a pulse sequence at a repetition rate of 52.8 MHz using an electro-optic intensity modulator driven by an AWG (Keysight M8196A, 32-GHz analog bandwidth). The modulation pulse profile is chosen to be a ~850-ps-wide Gaussian pulse. This pump is then amplified using an EDFA (Thorlabs EDFA300P) followed by an optical BPF (Santec OTF-350). A 20% tap is sent to the control unit for active interferometric phase stabilization (PHASE.FIX, Ki3 Photonics Technologies), further described below in the “Visibility measurements” section. The pump power is adjusted using a microelectromechanical system–based VOA (Thorlabs VOA1550PA) controlled by a programmable dc voltage supply. Specifically, pump powers of 0.5, 1.0, 2.0, 4.0, 6.3, and 10.0 mW are sent to the source to obtain mean photon numbers $\bar{n}$ of (2.97 ± 0.04) × 10^−3^, (4.25 ± 0.03) × 10^−3^, (8.84 ± 0.06) × 10^−3^, (23.02 ± 0.07) × 10^−3^, (48.0 ± 0.1) × 10^−3^, and (98.5 ± 0.2) × 10^−3^, respectively. The mean photon number per pulse is defined as $\bar{n}=\frac{S_{s}S_{i}}{C_{s,i}N_{p}}$, where $S_{s}$ and $S_{i}$ are the single-photon counts recorded for the signal and idler, respectively, $C_{s,i}$ is the number of coincidences, and $N_{p}$ is the total number of pump pulses sent during the duration of the measurement (*80*). The pump then travels through a polarizing optical beam splitter to ensure the transmission of a single linear polarization to excite the MRR resonance. A polarization-maintaining high-rejection (90 dB) BPF (Lightwave 2020) centered at the international telecommunication union (ITU) channel H34.5 and with a 200-GHz bandwidth is used to remove the noise from the pump beam before entering the MRR (Ki3 Photonics Technologies). After SFWM, the pump is separated from the generated photon pairs using a high-rejection notch filter with the same technical specifications, where the pump travels through the pass port within a 200-GHz bandwidth. In contrast, the generated biphotons across other frequencies travel through the reflect port. Noise generated by an attenuated EDFA is filtered down to a 9-GHz bandwidth for all measurements, except for the results shown below in Fig. 5 and figs. S4 and S14 to S16, and then injected into the biphotons using a 99/1 coupler. For all measurements of the source, the waveshaper bandwidth for demultiplexing the signal and idler photons just before detection is set to 12.5 GHz, while it is set to 70 GHz for the cases measured at the output of the denoising module with the PM on and PM off.

**Table 1.** Summary of experimental parameters. FSR, free spectral range.

Characteristic time/delay/bandwidth	Value
FSR of the MRR	100 GHz
Resonance line width of the MRR	~400 MHz
Single-photon pulse width $\approx 1/∆\nu_{\text{MRR}}$	~2.7 ns
Pump repetition rate	52.77 MHz
Pump bandwidth	1.2 GHz
Spacing between qTAI pulses $t_{q}$	2.479 ns
Theoretically expected width of qTAI pulses $t_{p}$	32.2 ps
Measured qTAI pulse full width at half maximum	60 ps
Target enhancement or compression factor, $q=t_{q}/t_{p}$	77
SNSPD timing jitter	50 ps

### Data analysis

To find the counts located within the TAI peaks, all the measured events are plotted as a function of the counts versus time (single-photon histogram) within the duration of each clock cycle, given by the repetition rate of the pumping scheme. The data with the qTAI activated and without any injected noise are first analyzed, and the obtained time windows are retained as the noise injection rate is increased (thus, the location of the peaks is known even in the very noisy cases). The peak locations and their FWHM are found by running a peak find algorithm based on the peak height and prominence. The width of each peak for the analysis is given by ~1.5× the obtained FWHM (~60 ps), resulting in a 93-ps-wide selection window, on average. For each mean photon number setting, this algorithm is used to find the temporal region of analysis. For the source measurements and those with the denoising module turned off, the counts present in the entire range of the qTAI peaks are considered (i.e., from the first index of the first TAI peak to the last index of the last TAI peak).

### Visibility measurements

The scheme that we use to demonstrate quantum interference is based on sequential time-bin entanglement (*64*, *65*). In this scheme, a train of pulses with a fixed phase relation is sent to a nonlinear crystal to generate biphotons in a superposition of time modes. As shown in fig. S2B, the output of the biphoton source is connected to an isolator to prevent residual light from the pump used to stabilize the interferometer module from entering the MRR. It is then sent to one of the arms of the stabilized fiber-based Michelson interferometer (Ki3 Photonics Technologies). The relative delay of the interferometers is set to the period of the pump pulse train, 18.95 ns, and its relative phase is tunable and can be stabilized on set points for signal integration. The biphotons are then retrieved from the other port of the interferometer. A second notch filter is used to route the biphotons from the input to the reflect port. We note that the pulses from the pump reference field are temporally separated from the biphotons (i.e., shifted by 9.7 ns with respect to each other) to minimize noise in the interferometric measurements (*81*). The visibility V is obtained following the relation $V=\frac{CC_{\text{max}}-CC_{\text{min}}}{CC_{\text{max}}+CC_{\text{min}}}$, where $CC_{\text{max}}$ and $CC_{\text{min}}$ are the maximum (0, π, and 2π) and minimum (π/2 and 3π/2) coincidence rates of the biphoton correlations from the interference measurement, respectively (*82*).

### Quantum state tomography measurements

The quantum state tomography measurements are implemented following the scheme described in (*67*), which we briefly review here. The principle relies on reconstructing the density matrix from four separate measurement settings, where the signal and idler photons go to separate interferometers with the appropriate phases (depicted as $\theta_{s}$ and $\theta_{i}$ in fig. S3). This allows us to obtain the 16 projection measurements corresponding to the entries of the 4 × 4 density matrix describing the entangled photon pair

$$n_{\nu }=C\left\langle \psi_{\nu }|\hat{\rho }|\psi_{\nu }\right\rangle$$

where $n_{\nu }$ corresponds to the number of coincidences for a projection on state $\psi_{\nu }$ of the density matrix $\hat{\rho }$ and C denotes a proportionality constant depending on the experimental setup. Specifically, considering a two-level time-bin scheme, the state of the signal and idler photons at the output of the interferometer is described by a triple-peak distribution, as shown in fig. S3. It is composed of counts projected in the side peaks, corresponding to the time basis states ∣1⟩ and ∣2⟩ and counts projected in the center bin, which depicts the state in the energy basis ∣E⟩. Applying a phase θ projects the energy basis onto the state $\frac{|1\rangle +e^{-i\theta }|2\rangle }{\sqrt{2}}$, such that using a phase of θ=0 projects to the state $|+\rangle =\frac{|1\rangle +|2\rangle }{\sqrt{2}}$, while a phase of θ=−π/2 projects to the state $|L\rangle =\frac{|1\rangle +i|2\rangle }{\sqrt{2}}$. For a given setting of $\theta_{s}$ and $\theta_{i}$, there are a total of nine accessible coincidence measurements (i.e., distinct combinations) between each peak pair of the triple-peak distributions. Measuring these counts for the appropriate four different phase settings allows us to gather the projections needed to reconstruct an estimate of the density matrix (*66*). Specifically, the four phase settings of the signal and idler photons $\left\{\theta_{s},\theta_{i}\right\}$ are {0,0}, {−π/2,0}, {0,−π/2}, and {−π/2,−π/2}, respectively. A physically legitimate density matrix that accounts for experimental inaccuracies and statistical fluctuation is then reconstructed using a maximum likelihood estimate algorithm (*66*, *67*).

The measured count rates for each projection are shown in table S1, where the count rates have been normalized to account for the intrinsic losses (i.e., total measured counts over the integration time of one phase setting measurement). We note that some of these projections are associated with inherent losses due to lost counts from the unmeasured interferometer output. However, these lossy projections are characterized in more than one of the four measurement settings (e.g., projections $n_{1}$ to $n_{4}$ are present in all measurements), such that this intrinsic loss is automatically compensated for when measuring all four phase settings. To access the time basis, the QST measurements are modified by using a conventional double-pulse pumping scheme, composed of two pulses separated by 18.95 ns, with a repetition rate of 17.59 MHz. See fig. S2C for the experimental setup. For each setting, the integration time for the source measurement is 11 min, while it is 17 min for the measurement at the output with the PM-off and PM-on cases.

## Supplementary Materials

### This PDF file includes:

Supplementary Texts 1 to 6 Figs. S1 to S16 Table S1 References
