# Supplementary material for: Quantum state revival via coherent energy redistribution
Source: Sci Adv. 2026 Jan 30;12(5):eady8981. doi: 10.1126/sciadv.ady8981 (PMC12857731; doi:10.1126/sciadv.ady8981)
Supplement: Supplementary file 1 — Supplementary Texts 1 to 6 Figs. S1 to S16 Table S1 References [file sciadv.ady8981_sm.pdf]

Science Advances
AAAS

Supplementary Materials for

Quantum state revival via coherent energy redistribution

Benjamin Crockett *et al.*

Corresponding author: Benjamin Crockett, Benjamin.Crockett@inrs.ca; José Azaña, Jose.Azana@inrs.ca

Sci. Adv. **12**, eady8981 (2026)
DOI: 10.1126/sciadv.ady8981

This PDF file includes:

Supplementary Texts 1 to 6
Figs. S1 to S16
Table S1
References

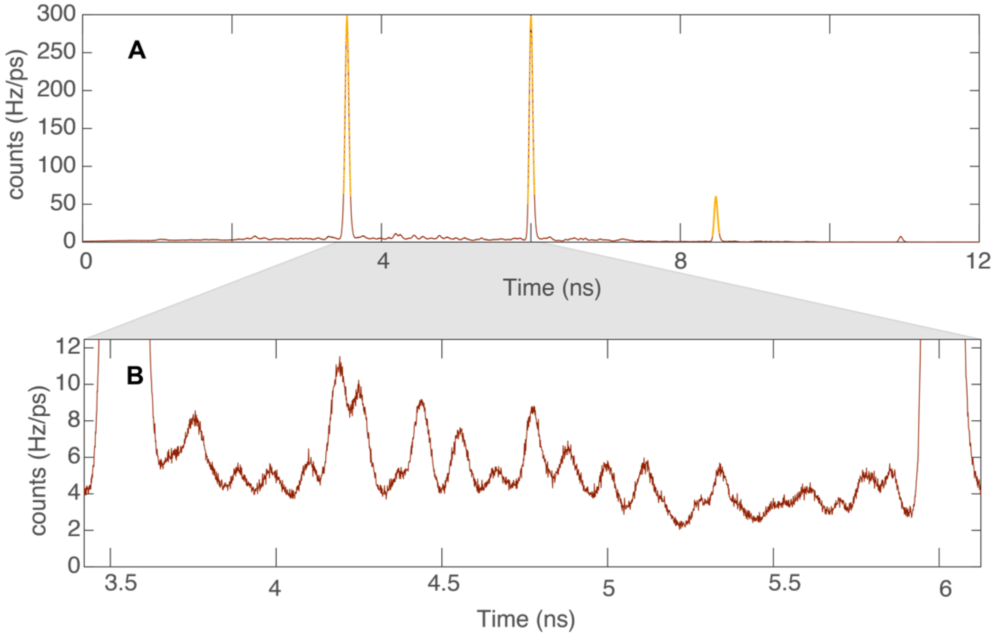

Fig. S1. Satellite peaks from imperfect energy redistribution. Due to experimental imperfections in the phase manipulation, a portion of the photon counts remains between the peaks rather than being focused into the qTAI peaks. (**A**) qTAI signal, with a mean photon number of 0.099 and no noise injection. An important step in implementing the qTAI consists of optimizing the phase manipulations to maximize the correlated counts within each qTAI peak, effectively reducing the photon loss associated with the satellite peaks presented in **B,** which depicts a zoomed-in version of A between the peaks.

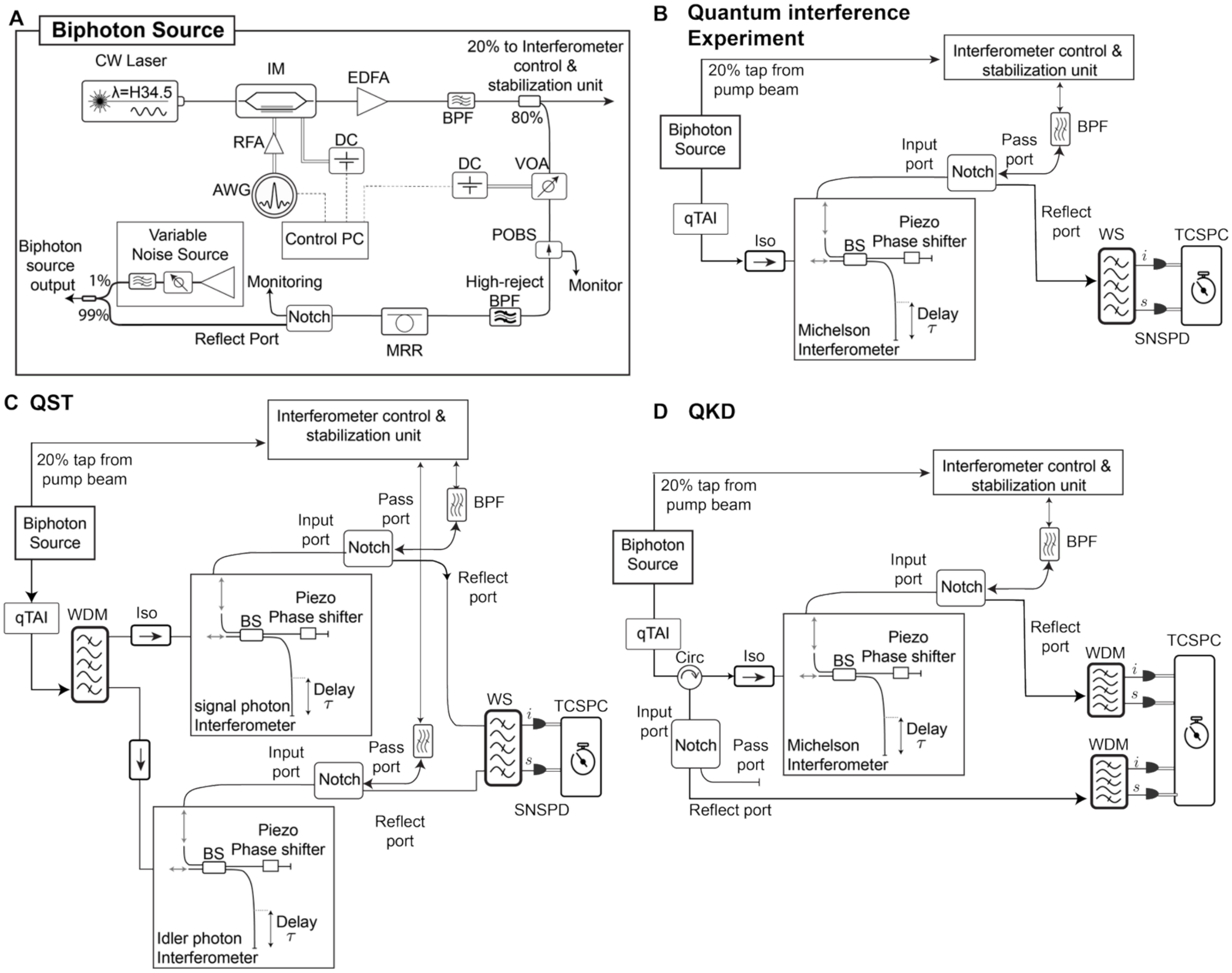

Fig. S2. Experimental setups. (**A**) Biphoton generation setup based on carved continuous wave (CW) pumping. IM: Intensity modulator; EDFA: Erbium-doped fiber amplifier; DC: Direct current voltage supply; RFA: Radio-frequency amplifier; AWG: Arbitrary waveform generator; BPF: Bandpass filter; VOA: Variable optical attenuator; MRR: Microring resonator; POBS: Polarizing optical beam splitter; OSA: Optical spectrum analyzer. (**B**) Experimental setup for quantum interference measurements; WS: Waveshaper (programmable multi-port optical filter); SNSPD: Superconducting nanowire single-photon detector; TCSPC: Time-correlated single-photon counter; Iso: Isolator. (**C**) Experimental setup for quantum state tomography (QST) measurement. (**D**) Experimental setup for quantum key distribution (QKD) measurement.

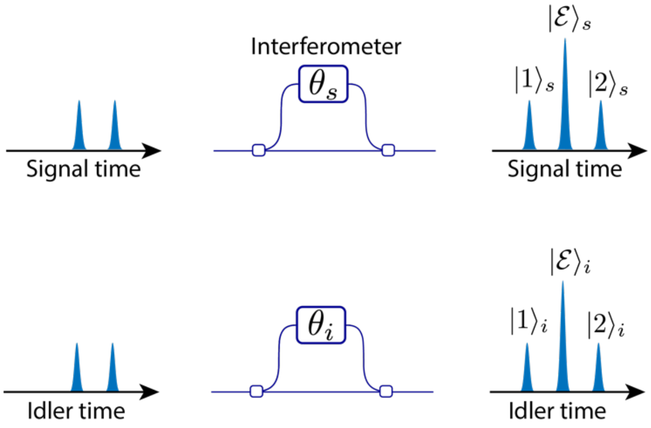

Fig. S3. Projection measurements for quantum state tomography.

Table S1: Count rates (in Hz) from the quantum state tomography experiment

Projection		Noiseless case			200 kHz noise injection		
		Source	PM off	PM on	Source	PM off	PM on
n_1	$\|1\rangle_s\|1\rangle_i$	26.6	2.73	1.18	40	4.89	1.28
n_2	$\|1\rangle_s\|2\rangle_i$	0.647	0.0975	0.0237	14.4	2.38	0.112
n_3	$\|2\rangle_s\|1\rangle_i$	0.515	0.0719	0.0227	14.1	2.4	0.0995
n_4	$\|2\rangle_s\|2\rangle_i$	25.9	2.47	1.05	38.8	4.83	1.17
n_5	$\|2\rangle_s\|+\rangle_i$	13.1	1.24	0.55	20.1	2.57	0.612
n_6	$\|1\rangle_s\|+\rangle_i$	14	1.37	0.63	21.4	2.63	0.684
n_7	$\|+\rangle_s\|+\rangle_i$	25.2	2.46	1.06	29.4	3.2	1.13
n_8	$\|L\rangle_s\|+\rangle_i$	8.98	0.943	0.394	13.2	1.53	0.38
n_9	$\|L\rangle_s\|1\rangle_i$	13.5	1.35	0.578	21.4	2.8	0.676
n_{10}	$\|L\rangle_s\|2\rangle_i$	13.4	1.28	0.578	21	2.59	0.638
n_{11}	$\|L\rangle_s\|L\rangle_i$	0.939	0.152	0.0549	5.34	0.864	0.0933
n_{12}	$\|1\rangle_s\|L\rangle_i$	13.9	1.37	0.591	21.4	2.71	0.648
n_{13}	$\|2\rangle_s\|L\rangle_i$	12.8	1.29	0.581	20.1	2.51	0.646
n_{14}	$\|+\rangle_s\|L\rangle_i$	9.75	0.993	0.419	14.2	1.75	0.473
n_{15}	$\|+\rangle_s\|1\rangle_i$	13.7	1.34	0.587	21.5	2.75	0.63
n_{16}	$\|+\rangle_s\|2\rangle_i$	13.3	1.32	0.598	21.2	2.74	0.579
Fidelity		0.921	0.895	0.906	0.618	0.523	0.858

1. Talbot array illuminator theory

Here we give a theoretical description of the redistribution process of the joint temporal distribution of entangled photon pairs using the Talbot array illuminator. We first review the classical implementation for 1D classical signals and then adapt the formalism to the 2D description of entangled photon pairs.

Note that the photons pairs in the results shown here are nearly uncorrelated in the time-frequency mode, since they are produced via SFWM in an MRR, with the pump bandwidth being larger than the resonance width. Consequently, the time-scale of the correlations is on the order of the pump repetition rate (~50 MHz in this experiment) which is much slower than the modulation bandwidth of the TAI phases (~20 GHz). Therefore, the time-frequency modes transform similarly to spatial images. If instead, the correlation dynamics were on the order of the modulation bandwidth, then a frequency-to-time mapping effect would be induced (*53*, *83*), a description that falls outside the scope of the present paper.

To describe the peak formation caused by the TAI, we must first obtain the expression for the Talbot phases that result from the temporal Talbot effect. Thus, consider a periodic 1D (or classical) signal with period t_q as

$$e(t) = \sum_{k=-\infty}^{\infty} \delta\left(t - kt_q\right) * a(t),$$

where $*$ denotes convolution, and $a(t)$ is the temporal profile of a single pulse in the periodic sequence. As described by the temporal Talbot effect, a periodic signal, without initial pulse-to-pulse phase variation, will be reimaged with its period divided by an integer q after a dispersive propagation described by a transfer function $H(\omega) = \exp(-i\ddot{\phi}\omega^2/2)$, with the second-order dispersive coefficient satisfying $\ddot{\phi} = \frac{p}{q}\frac{t_q{}^2}{2\pi}$, where p is an integer co-prime with q (*84*). This is the so-called fractional Talbot effect. It can be shown that in the temporal domain (*52*), the waveform can be expressed as

$$e'(t) = \delta\left(t - e_{pq}\frac{t_q}{2}\right) * \sum_{k=-\infty}^{\infty} \delta\left(t - kt_q\right) * \frac{1}{\sqrt{q}}\sum_{n=0}^{q-1} e^{i\sigma_\phi \xi_n}\delta\left(t - n\frac{t_q}{q}\right) * a(t), \qquad \textbf{(S1)}$$

where e_x represents the parity of x ($e_x = 1$ if x is odd, and $e_x = 0$ otherwise). The convolution with the last delta function depicts that the sequence periodicity is divided by a factor q, and the pulses have a q-periodic pulse-to-pulse phase variation satisfying

$$e^{i\sigma_\phi \xi_n} = \frac{1}{\sqrt{q}}\sum_{m=0}^{q-1} e^{-i\pi\sigma_\phi\frac{p}{q}(1+qe_q)m^2}\, e^{i2\pi\frac{nm}{q}} = e^{i\sigma_\phi \xi_0}\exp(i\pi\sigma_\phi\frac{s}{q}n^2),$$

where $\sigma_\phi = \pm 1$ corresponds to the sign of $\ddot{\phi}$, $e^{i\sigma_\phi \xi_0}$ is an overall phase offset, and the integer s is related to p and q as $sp = 1 + qe_q (mod\ 2q)$. The second delta function in Eq. (S1) indicates that this unit repeats over infinity. The first delta function depicts a half-period shift depending on the parity of the integers p and q. We refer the reader to Ref. (*52*) for a thorough description of the phases governing the Talbot effect.

The idea behind the TAI is to apply the inverse phase observed from the temporal Talbot effect, so that after the corresponding dispersive propagation, q adjacent bins are coherently summed together. To see this, consider the situation where $a(t)$ in Eq. (S1) is a square pulse of width t_q/q, with a period of t_q. After a dispersive propagation characterized by the integer q, the input pulses are replicated adjacent to one another, such that the output waveform consists of a continuous wave having an intensity q times lower than the input pulses and with a phase variation following $e^{i\sigma_\phi \xi_n}$. Thus, by implementing the TAI, we are effectively doing the inverse operation by imposing the phase variation of a fractional Talbot image on a continuous waveform, such that dispersive propagation redistributes the energy into peaks of width t_q/q separated by t_q.

Therefore, to describe the TAI, consider that the input signal is a continuous wave with a discretely-modulated temporal phase as

$$e(t) = \sum_{k=-\infty}^{\infty} \delta(t - kt_q) * \sum_{m=0}^{q-1} \exp(-i\sigma_\phi \xi_m) \operatorname{rect}\left(\frac{tq}{t_q} - m\right),$$

where $\operatorname{rect}(x) = 1$ if $|x| < \frac{1}{2}$ and $\operatorname{rect}(x) = 0$ otherwise. Then, using Eq. (S1) and omitting the factor $\delta(t - e_{pq}T)$ since it only accounts for an overall shift, we determine the output waveform as

$$e'(t) = \sum_{n=-\infty}^{\infty} \sum_{m=0}^{q-1} \exp\big(i\sigma_\phi(\xi_n - \xi_m)\big) \operatorname{rect}\left(\frac{tq}{t_q} - n - m\right)$$

Which, owing to the autocorrelation property of the Talbot phases (*52*), can be expressed as

$$e'(t) = \sqrt{q} \sum_{n=-\infty}^{\infty} \operatorname{rect}\left(\frac{tq}{t_q} - nq\right). \qquad \textbf{(S2)}$$

Thus, we find that the output waveform is a series of square pulses of width $\frac{t_q}{q}$, repeating with a period t_q.The TAI has a similar effect on an uncorrelated quantum state, as we show here. let us consider a generic biphoton quantum state, where the time scale of the correlations are much slower than the TAI phases we wish to impose on it, as discussed above. We expressed it in the time domain as (*49*)

$$|\psi(t_s, t_i)\rangle = \iint dt_s dt_i\, f(t_s, t_i)\, \hat{a}_s^\dagger(t_s) \hat{a}_i^\dagger(t_i)|0\rangle$$

Where s and i correspond to the signal and idler photon states, $|0\rangle$ is the vacuum state, $\hat{a}_s^\dagger(t_s)$ and $\hat{a}_i^\dagger(t_i)$ are the creation operators for signal and idler photons, respectively, and $f(t_s, t_i)$ is the joint temporal distribution of the biphoton state (*49*). Below we show how the joint temporal distribution is then modified using the qTAI.

Again, to find the Talbot phases needed for the TAI, we first consider the equivalent temporal Talbot effect for a periodically repeating uncorrelated state, with a biphoton state characterized by a joint temporal distribution as

$$f(t_s, t_i) = \sum_{n,m=-\infty}^{\infty} \delta\left(t_s - nt_q\right) * \delta\left(t_i - mt_q\right) * \alpha(t_s, t_i),$$

where $\alpha(t_s, t_i)$ is a joint distribution that extends over a duration less than $\frac{t_q}{q}$ for both signal and idler photons. We observe that after group-velocity dispersion under a fractional Talbot condition, the state evolves as

$$f'(t_s, t_i) = \delta\left(t_s - e_{pq}\frac{T}{2}\right) * \delta\left(t_i - e_{pq}\frac{T}{2}\right) * \sum_{k_s=-\infty}^{\infty} \delta\left(t_s - kt_q\right) * \sum_{k_i=-\infty}^{\infty} \delta\left(t_i - kt_q\right)$$
$$* \frac{1}{\sqrt{q}} \sum_{n_s=0}^{q-1} e^{i\sigma_\phi \xi_n} \delta\left(t_s - n_s \frac{T}{q}\right) * \frac{1}{\sqrt{q}} \sum_{n_i=0}^{q-1} e^{i\sigma_\phi \xi_n} \delta\left(t_i - n_i \frac{T}{q}\right) * \alpha(t_i, t_s), \quad \textbf{(S3)}$$

and so we obtain the two-dimensional phase distribution of the fractional Talbot image, such that we can define a single unit cell of the needed phase mask over a duration t_q for both the signal and idler times as

$$w(t_s, t_i) = \sum_{n_s=0}^{q-1} \sum_{n_i=0}^{q-1} \exp\left(i(\xi_{n_s} + \xi_{n_i})\right) \operatorname{rect}\left(\frac{t_s}{t_q/q} + \frac{t_i}{t_q/q} - n_s - n_i\right),$$

Now that we have the equation for the phase mask needed for the qTAI, we can describe the transformations leading up to the energy redistribution of the joint distribution. We assume that fastest variations of the joint temporal distribution $f(t_s, t_i)$ of the input quantum state is continuous and extends over a duration much larger that the phase modulation period t_q, or equivalently, that the spectral width of the quantum state is much narrower than the spectral comb spacing of the temporal phase modulation $\sim \frac{1}{t_q}$ on time scales of t_q. After temporal phase modulation by the obtained Talbot phases, we obtain:

$$f'(t_s, t_i) = f(t_s, t_i) \left(\sum_{n_s=0}^{\infty} \delta\left(t_s - n_s t_q\right) * \sum_{n_i=0}^{\infty} \delta\left(t_i - n_i t_q\right) * w(t_s, t_i)\right). \quad \textbf{(S4)}$$

The state is then assumed to undergo dispersive propagation under a fractional condition. The resulting joint temporal distribution can then be calculated by inserting Eq. (S4) into (S3), leading to the following expression (omitting again the terms as $\delta\left(t - e_{pq}\frac{T}{2}\right)$):

$$f'(t_s, t_i) = \sum_{m_s, m_i=-\infty}^{\infty} \sum_{n_s, n_i=0}^{q-1} \exp\left(i\sigma_\phi\left(\xi_{n_s} - \xi_{m_s}\right)\right) \exp\left(i\sigma_\phi\left(\xi_{n_i} - \xi_{m_i}\right)\right)$$
$$\times \operatorname{rect}\left(\frac{t_s}{t_q/q} - n_s - m_s\right) \operatorname{rect}\left(\frac{t_i}{t_q/q} - n_i - m_i\right) f(t_s, t_i) \quad \textbf{(S5)}$$

Applying the autocorrelation property of the Talbot phases, Eq. (S5) can be simplified to

$$f'(t_s, t_i) = q \sum_{m_s, m_i=-\infty}^{\infty} \mathrm{rect}\left(\frac{t_s q}{T} - m_i q\right) \mathrm{rect}\left(\frac{t_i q}{T} - m_i q\right) f(t_s, t_i).$$

As predicted, the joint temporal distribution consists of a series of peaks within an envelope shaped by the input joint temporal distribution profile $f(t_s, t_i)$. This represents a quantum analogue to Eq. (S2), where the joint distribution of the photons are transformed into peaks, maintaining the slowly varying correlation properties. Consistently with the descriptions provided in the main text, the noise reduction occurs because an incoherent field, such as incoherent stochastic noise affecting the biphoton state, does not undergo the coherent summation of Eq. (S5). This field will instead spread out following the frequency-to-time mapping effect described in the main text, leading to an effective discrimination of this incoherent noise context with respect to the coherent quantum state function.

2. Schmidt number enhancement

By simultaneously processing multiple channels of the MRR, the noise mitigation from the qTAI can recover the spectral correlations of a deteriorated qubit state. In this case, we use an MRR with an FSR of 500 GHz, and a line width of ~200 MHz. We note that to process multiple resonances at once, the FSR of the MRR should be larger than the phase modulation bandwidth of the qTAI to avoid crosstalk between channels. When demultiplexing the signal and idler photons, the waveshaper bandwidth is set to 60 GHz for each, for both the denoising off and denoising on cases. In this case, the injected noise is not filtered before being injected into the system, such that it also has a bandwidth of 60 GHz.

We select the first and third channels (i.e., first and third signal-idler pairs), and describe the dimensionality of this state through the Schmidt number. Ideally, correlations are present only between pairs of channels with equal probability, such that a Schmidt number of 2 should be obtained when considering two signal-idler modes.

Figure S4A shows the time-of-arrival map between all signal-idler combinations with the denoising turned off, including those from different channels (i.e., i_1-s_3 and i_3-s_1). This representation is obtained by simply delaying the different channels with respect to each other. The coincidences can thus be mapped into a 2x2 matrix to get the representation shown in Fig. S4B. The various values of these bins correspond to the raw coincidences $CC_{i,s}$, which form the joint spectral intensity (JSI) of this 2-dimensional subspace of the MRR.

$$JSI = \begin{bmatrix} CC_{i_1,s_1} & CC_{i_1,s_3} \\ CC_{i_3,s_1} & CC_{i_3,s_3} \end{bmatrix}$$

The Schmidt number is obtained from the joint spectral amplitude (JSA) of the system, which is a complex-valued entity. Thus, by simply approximating the JSA as the square root of the JSI, a lower bound for the Schmidt number can be obtained (*54*). Therefore, to calculate the Schmidt number, we normalize the JSI such that the sum of its entries is equal to 1, and we find the approximated JSA by taking the square root. The Schmidt eigenvalues λ_n can be obtained by singular value decomposition of this JSA, and the Schmidt number can then be calculated as $K = (\sum \lambda_n^2)^{-1}$.

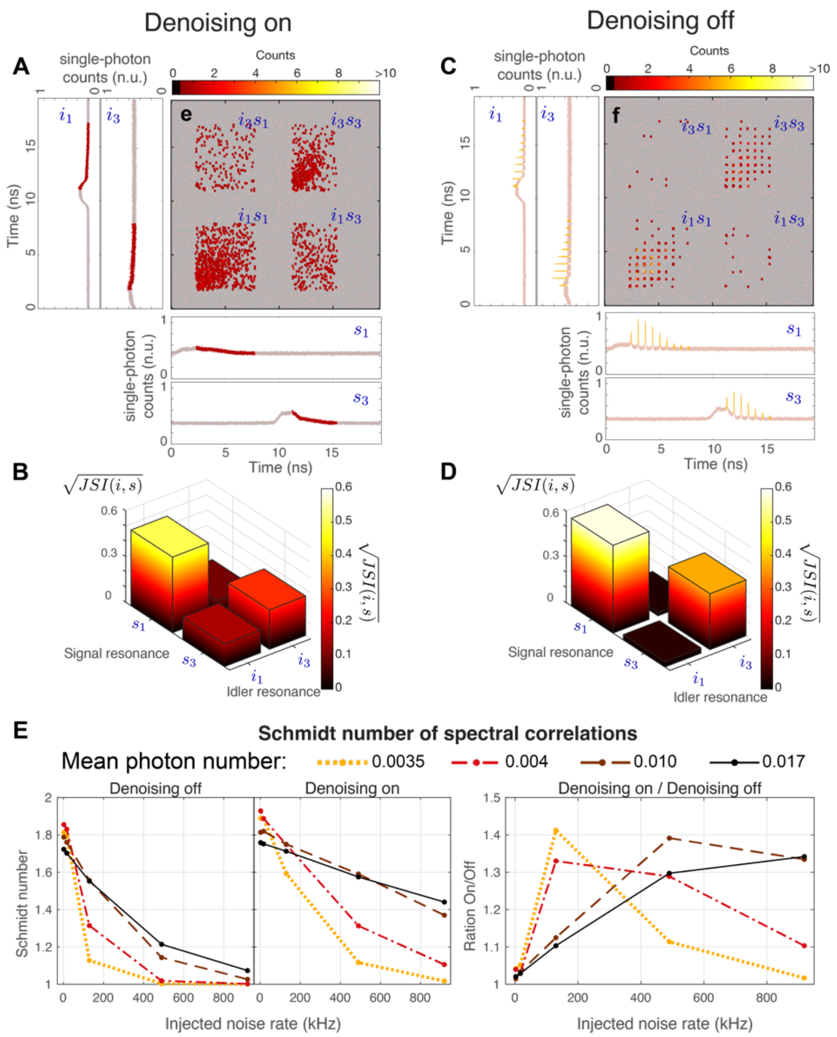

Fig. S4. Effect of qTAI on the Schmidt number. (**A**)To obtain a time-mapped representation of the JSI, a delay is applied to each channel separately during post-processing. (**B**) The counts are then grouped into a 2x2 matrix, from which a lower bound for the Schmidt number is obtained by singular value decomposition. (**C**), (**D**) Same as above, but with the denoising module activated. (**E**) Analysis for different mean photon numbers and noise injection rates, showing the Schmidt number for denoising off, denoising on, and the ratio of the Schmidt numbers with either denoising on/denoising off.

In the case of denoising off, Figs. S4A, B, a low Schmidt number of 1.1 is found, accounting for the large number of accidental coincidences along the anti-diagonal. We then process the quantum state using a qTAI with $q = 14$ and a peak widths of 47.8 ps using a dispersive line providing a total dispersion of $\ddot{\phi} = -5{,}082$ ps^2. Activating the denoising module leads to the results shown in Fig. S4C, D with a much-improved correlation along the diagonal, giving a Schmidt number of 1.6. This analysis is carried out for different mean photon numbers and noise injection rates in Fig. S4E, showing an improvement for all cases considered. The quoted injected noise rates on the x-axis of Fig. S4E correspond to those measured for the PM off case for the particular attenuation setting on the injected noise.

3. Extended results (Effect of synchronization, dark count mitigation, and proof-of-concept QKD experiment)

In the results shown up to here, the AWG employed for carving light pulses from the CW pump was synchronized with the temporal phase modulation signal, allowing the counts to be focused on the same temporal window for every repetition of the pump. Achieving such synchronization is feasible in practice, particularly in a time-bin entangled system relying on precise timing information to decode the quantum state. Nonetheless, we investigate here the effect of having a pump that is not synchronized with the phase modulation signal, which could find application in CW pumping schemes.

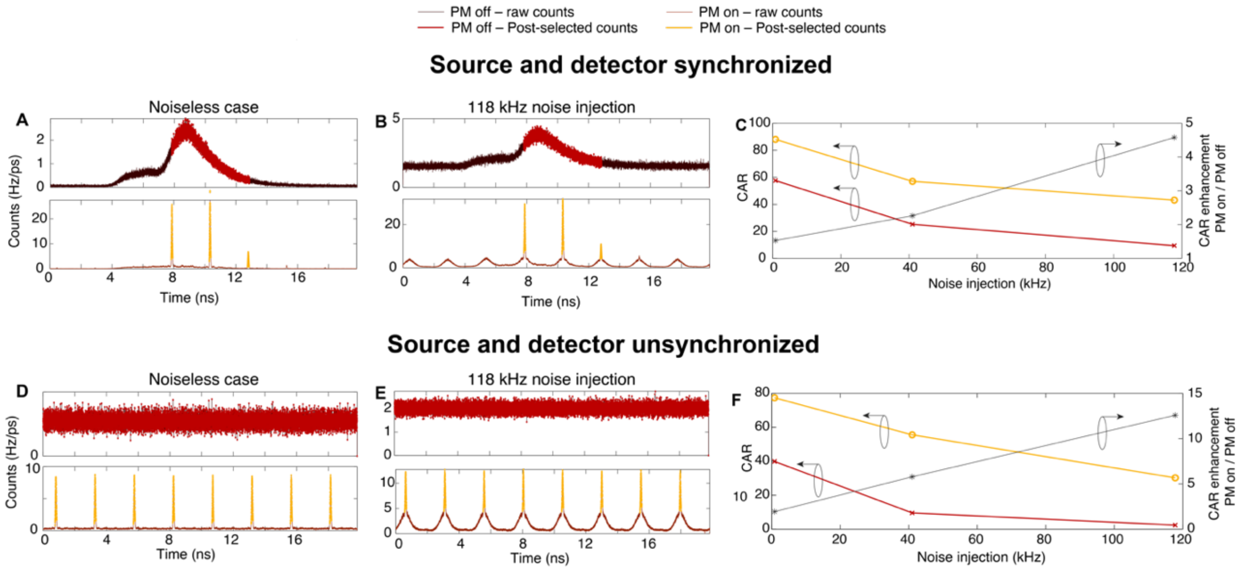

Fig. S5. CAR enhancement on an unsynchronized source. **A** Output counts with PM off and PM on, without injected noise. **B** Similar to A, but with a noise injection of 118 kHz. **C** CAR enhancement. **D-F** Same as the previous cases, but now without synchronization between the phase modulation and source. Notice the increase in the CAR enhancement in F, black trace (right axis).

Figure S5A shows the distribution of the signal photon without any injected noise for both PM off and PM on, where the pump is set to provide a mean photon number of 0.011. The single-photon histogram after injecting 118 kHz of noise (with a 9-GHz bandwidth) is presented in Fig. S5B. In Fig. S5C, we show the CAR enhancement. Now, we remove the synchronization between the pump and the phase modulation signal, as shown in Fig. S5D-F. Notice how the counts in the PM off case are uniformly distributed throughout the repetition period, whereas in the case of PM on, the qTAI peaks are still formed (albeit also present throughout, instead of only within the temporal extent of the biphoton). In turn, this allows for a significantly higher enhancement of the CAR (i.e., approximately 12.5 in Fig. S5F compared to ~4.6 in Fig. S5C). We note that postselection is also performed on the biphoton relative delay to account for noise counts that are recorded outside their coherence lengths. As expected, the CAR itself is lower due to the loss of synchronization. Still, these results demonstrate how the qTAI can allow for even higher enhancements when the source and detector are not synchronized. However, it should be noted however that a synchronization between the PM and detection system is needed; otherwise, the qTAI peaks would also be washed away, giving a uniform distribution. Given that biphoton processing through phase modulation occurs just before detection, this requirement should not, in practice, represent a challenge.

In Fig. S6, we show the unique capability of the qTAI system to denoise dark counts in detection systems. Here, the dark counts are increased by raising the bias current of the SNSPD. The qTAI allows for postselecting on the temporally redistributed narrow peaks of the coherent signal of interest, in turn allowing for the mitigation of the effect of dark counts that are spread out along the entire time axis. Thus, the qTAI could also be exploited to enhance the performance of small form factor single photon detectors, such as single-photon avalanche photodiodes (SPADs), which typically have a higher dark count rate than SNSPDs.

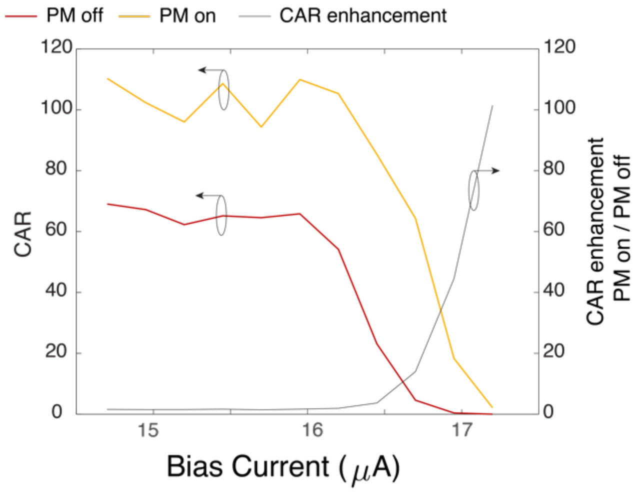

Fig. S6. Mitigation of dark counts as a function of detector bias current.

In Fig. S7, we demonstrate that the qTAI method would enable establishing a secure connection between two users via QKD in noisy conditions that would otherwise prevent the existence of a quantum-secure channel. For approximating the qTAI impact on a QKD link (setup presented in Fig. S2D), both outputs of the interferometer are utilized. Each of them is processed using wavelength division multiplexing (WDM) filters to separate the signal and idler photons, and then detected using four separate detectors (*85*). For simplicity, this proof-of-concept experiment employs a single interferometer for both signal and idler photons (*86*), whereas both users would have their own interferometer in a deployed QKD link, and the predicted SKR shown in the main text corresponds to Z-basis measurements, where the relative phase is set to 0, following the equation $SKR = r_{sift}[1 - f(e_b)H_2(e_b) - H_2(e_b)]$, where r_{sift} is the sifted key rate, taken as the coincidence rate, $e_b = (1 - V)/2$ is the quantum bit error rate defined in terms of the quantum interference visibility V, $f(e_b)$ is the error correction efficiency (set as 1.2), and $H_2(e_b)$ is the two-dimensional Shannon entropy function, $H_2(x) = -x\log_2(x) - (1 - x)\log_2(1 - x)$. In practice, to establish a secure key rate (rather than approximating it), the X-basis measurement would be needed for a portion of the measurements depending on the basis chosen. Typically, both X- and Z-bases are given equal probability, but more efficient approaches can give a larger probability to one basis over the other (*87*).

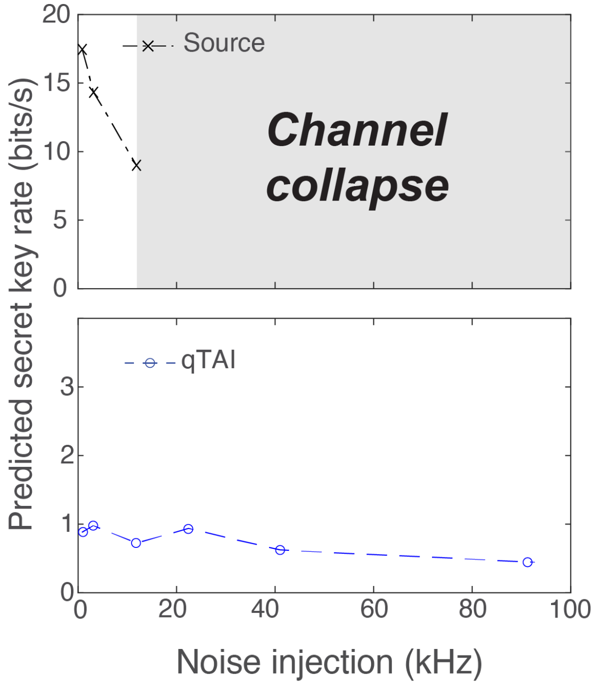

Fig. S7. Proof-of-principle QKD experiment. The qTAI retains a positive predicted secret key rate even at noise levels that would otherwise lead to the collapse of the channel. The secret key rate of the qTAI module output in the noiseless case could approach that of the source through further optimization of the insertion losses and phase modulation process.

4. CAR enhancement theoretical model

In this section, we analyze the noise mitigation mechanism of the energy redistribution process, showing the dependence of the CAR improvement on the enhancement factor q. We show how the noise mitigation process affects both coherent and incoherent noise components, and obtain a fit parameter that relates to properties of the noise source, such as its time and spectral distributions, which remain constant with varying noise injection rates and mean photon numbers. This model could be employed to investigate various types of noise produced in the generation or transmission of quantum states.

We follow here the effect of the noise bandwidth via frequency-to-time mapping and the detection resolution to derive the effective enhancement factor $\tilde{q}$, which is then numerically fit to the experimental data. Considering broadband noise (i.e., featuring a noise bandwidth $\Delta\nu_n \gg 1/t_p$), the enhancement factor is given by $q = t_q/t_p$. If the temporal resolution of the system broadens the TAI peaks to $\tilde{t}_p$, then the compression factor is decreased according to $\tilde{q} = t_q/\tilde{t}_p$. Here, this is taken as the mean width of the postselection window on the peaks, which is 93 ps. Additionally, if the noise covers a bandwidth $\Delta\nu_n$ smaller than $1/t_p$, then this noise is mapped onto the time domain following $\Delta t_n = 2\pi\ddot{\phi}\Delta\nu_n$, and we obtain a noise reduction factor $\tilde{q}_n = \Delta t_n/t_p$. In the experiments shown here, the noise has a bandwidth of 9 GHz, and the dispersive line provides a total dispersion $\ddot{\phi} = 12{,}758\ \text{ps}^2$, so the noise spans a temporal duration of ~720 ps, as explained in section *Experimental Implementation* of the main paper. The full width at half maximum of the noise distribution covers an interval of 666 ps and 864 ps for the signal and idler photons,

respectively, where the discrepancy results from third-order dispersion in the LCFBG. We thus take the mean value of 765 ps for calculating $\tilde{q}_n$. This gives a noise reduction factor $\tilde{q}_n = \Delta t_n/\tilde{t}_p = 8.2$.

We first consider the accidental counts without noise injection. In this case, we make the approximation that the noise contribution in the single-photon counts of the signal and idler, p_s^0 and p_i^0, respectively, are dominated by coherent photon counts c_s and c_i,

$$\begin{aligned} p_s^0 &\approx c_s \\ p_i^0 &\approx c_i,. \end{aligned} \quad \textbf{(S6)}$$

such that the accidental counts can be expressed as

$$\begin{aligned} AC_0 &= p_i^0 \times p_s^0 \\ &= c_i \times c_s \approx c^2 \end{aligned}$$

In this latest expression, we have also assumed that $c = c_i = c_s$. This approximation is valid for high mean photon numbers (associated with more multiphoton generation events), since we are effectively neglecting the noise contributions from incoherent sources such as that generated by Raman scattering from the co-propagating pump field in the fiber (*88*). These accidental counts are composed of uncorrelated counts resulting from multiphoton events, pairs with a lost photon, etc. This type of "quantum noise" represents events that cover the same time and frequency distribution as the biphoton counts, however they are due to daughter photons originating from unmatched generation instances.

Now, we consider the case of incoherent noise injection (generated by the EDFA in the experiment) by expressing the accidental counts as (*59*)

$$\begin{aligned} AC &= p_i \times p_s \\ &= \left(\sqrt{AC_0} + \gamma x_n\right)^2 \\ &= AC_0 + \gamma^2 x_n^2 + 2\sqrt{AC_0}\gamma x_n, \end{aligned}$$

where the injected incoherent noise rate is given by x_n, and γ is a proportionality factor that relates the incoherent noise rate to the accidental counts. The CAR can now be expressed as

$$CAR = \frac{C_T}{AC_0 + \gamma^2 x_n^2 + 2\sqrt{AC_0}\gamma x_n}, \quad \textbf{(S7)}$$

where C_T is the true correlated count rate, defined from the measured coincidence CC and accidental AC count rates as $C_T = CC - AC$. Thus, we see that accidental counts arise from coincidences between either two coherent (first term in the denominator) or incoherent (second term in the denominator) noise counts, or a coherent with an incoherent noise count (third term in the denominator). The qTAI improves the CAR by ideally reducing the incoherent counts received by each single-photon detector by a factor of q, labelled here as $\tilde{q}_n$ to account for the non-idealities described above. The CAR can thus be expressed as

$$CAR_{qTAI} = \frac{C_T}{AC_0 + \gamma^2 x_n^2/\tilde{q}_n{}^2 + 2\sqrt{AC_0}\,\gamma x_n/\tilde{q}_n}. \qquad \textbf{(S8)}$$

Here, C_T is taken as the correlated count rate in the noiseless case (which remains approximately constant as the injected noise increases, see Fig. S12 A or S13B), while AC_0 is taken as the accidentals in the noiseless case for each mean photon number and phase modulation setting. For simplicity, we compare here the data at the output with the modulation turned on vs off (PM on vs. PM off). We perform a fit of Eqs. (7) and (8) as a function of the noise injection rates, with γ as an unknown, for four different mean photon numbers, shown in Fig. S8. The fit parameter γ is found to have a mean value of (2.0±0.1)$\times 10^{-5}$. The ratio of these fits also agrees with the enhancement factor of the measured CAR, Fig. S8C. This indicates that the proposed CAR mathematical model provides a good representation of the denoising principle behind the qTAI. Notice how a deviation from the model starts to appear at low mean photon numbers, since the approximation in Eq. (S6) assumes that the accidental counts without noise injection are dominated by coherent noise. However, incoherent noise sources such as Raman noise become increasingly significant at this low-power regime.

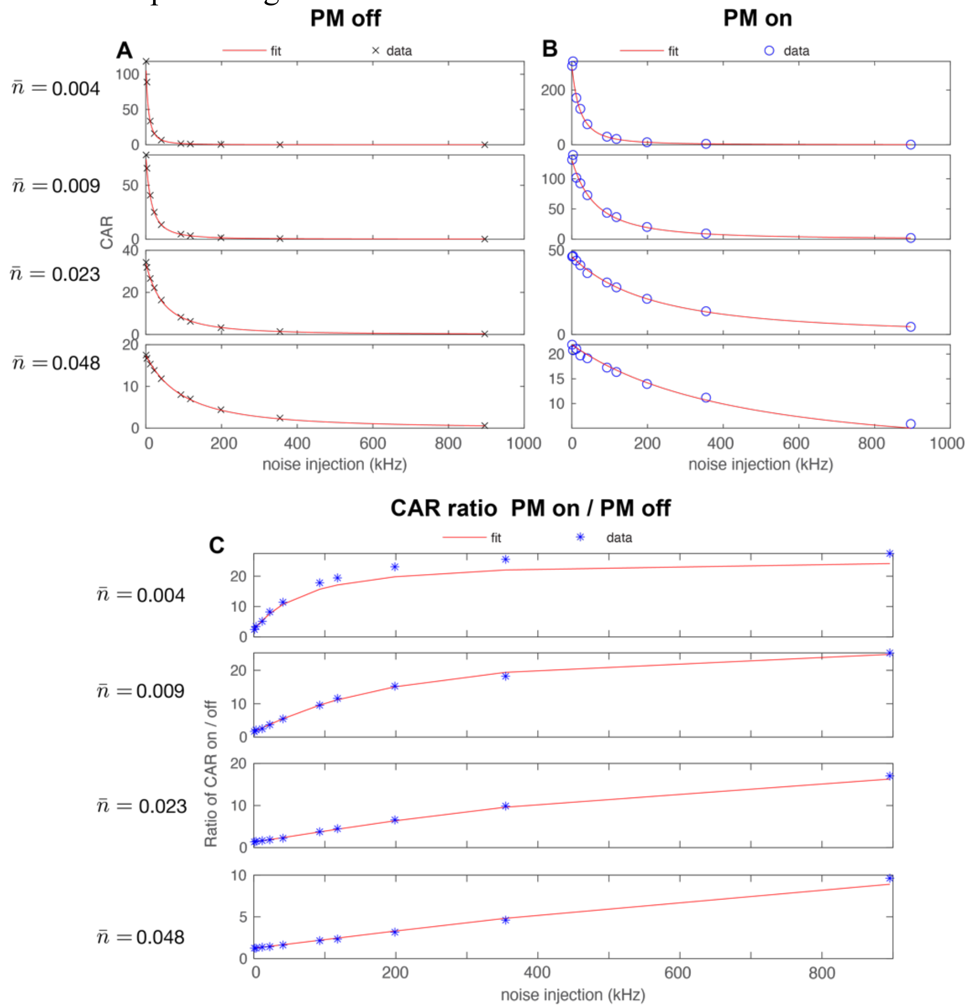

Fig. S8. Fit of the theoretical model to the measurement data describing the CAR enhancement. CAR data and fit with a noise bandwidth of 9 GHz, at the output of the qTAI system with the phase modulator turned off (A) and on (B). (**C**) CAR enhancement ratio, given by the ratio of the data shown in B and A. The data agree well with the fit, with a slight discrepancy at low mean photon numbers, where the approximation that multiphoton events dominate the accidental counts with noise injection becomes less accurate.

5. Histograms and further details on the CAR enhancement results vs mean photon number and noise injection rates (9-GHz noise bandwidth)

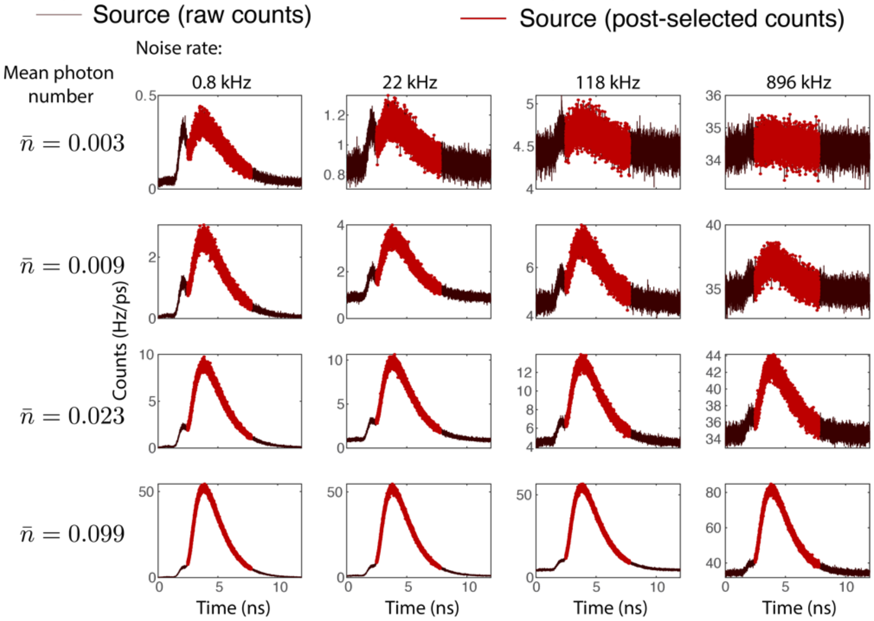

Fig. S9. Sample histograms of signal photons at the source. Mean photon numbers are shown increasing from top to bottom, and injected noise rates increasing from left to right. These results correspond to the experiments reported in the main text, with a noise bandwidth of 9 GHz.

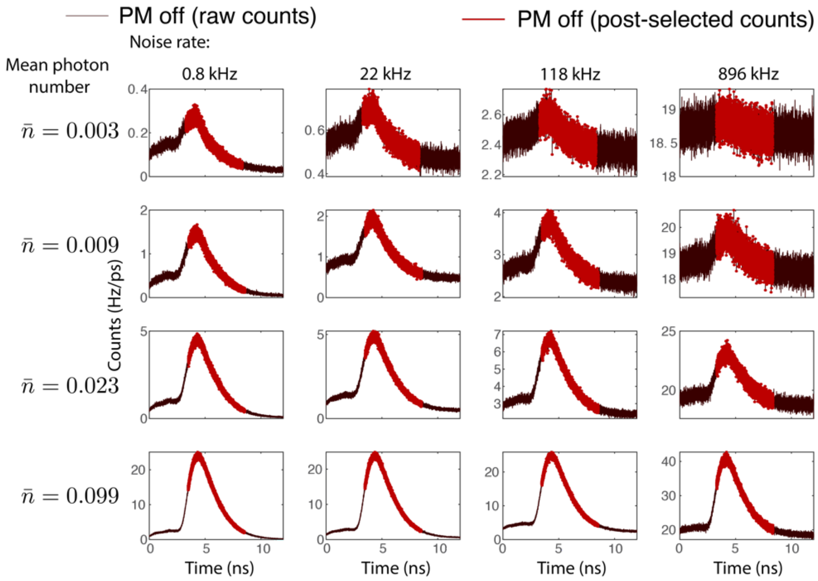

Fig. S10. Sample histograms of signal photons at the output of the qTAI module, with the phase modulation (PM) turned off. Mean photon numbers are shown increasing from top to bottom, and injected noise rates increasing from left to right. These results correspond to a noise bandwidth of 9 GHz.

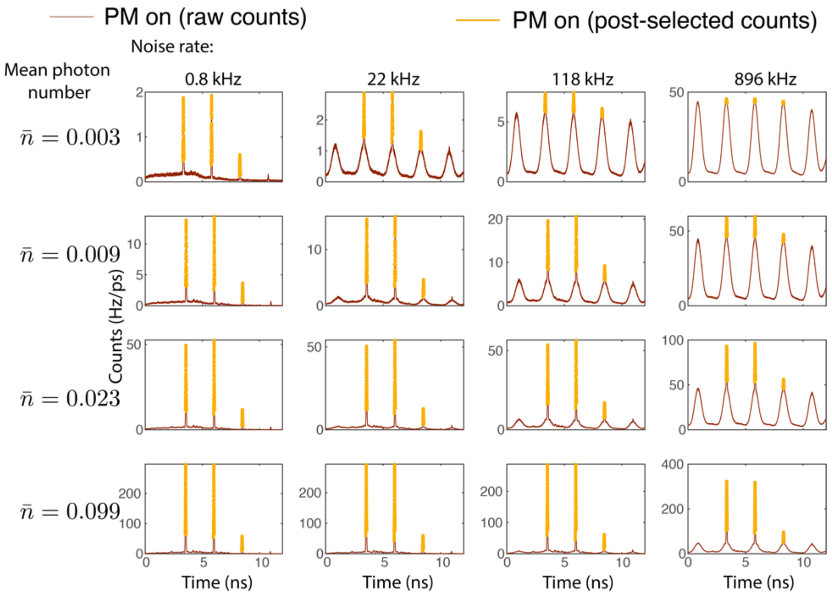

Fig. S11. Sample histograms of signal photons at the output of the qTAI module, with the phase modulation (PM) turned on. Mean photon numbers are shown increasing from top to bottom, and injected noise rates increasing from left to right. These results correspond to from the experiments reported in the main text, with a noise bandwidth of 9 GHz.

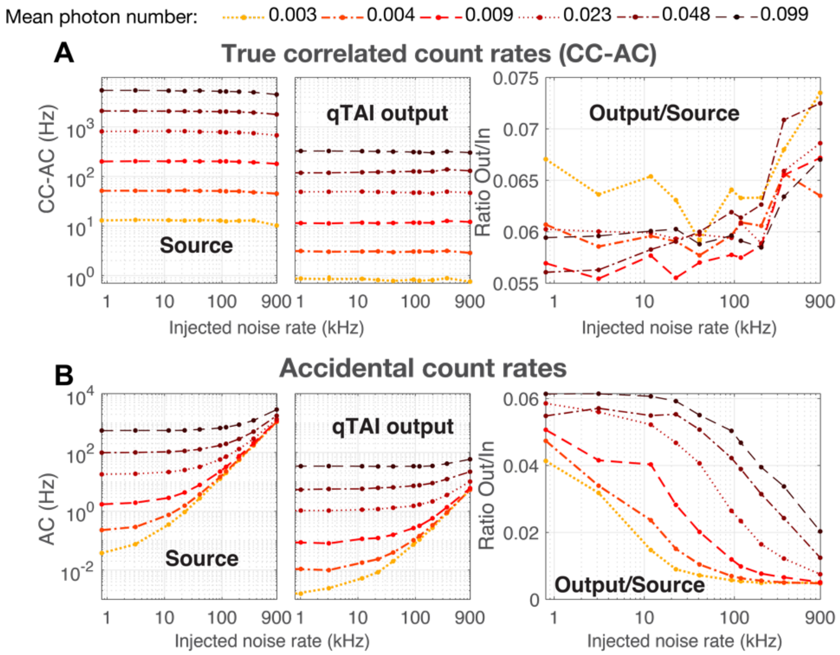

Fig. S12. CAR-related metrics for all cases considered in Fig. 4 of the main text, between the source and the output of the qTAI. (**A**) True correlated count rate $C_T = CC - AC$ for different mean photon numbers and injected noise rates. Notice how the true correlations are relatively constant for all noise injection rates at a given mean photon number setting. (**B**) There is a significant decrease in the accidental counts AC at the qTAI output due to the noise count discrimination allowed by the energy redistribution process, resulting in the observed increase of the CAR.

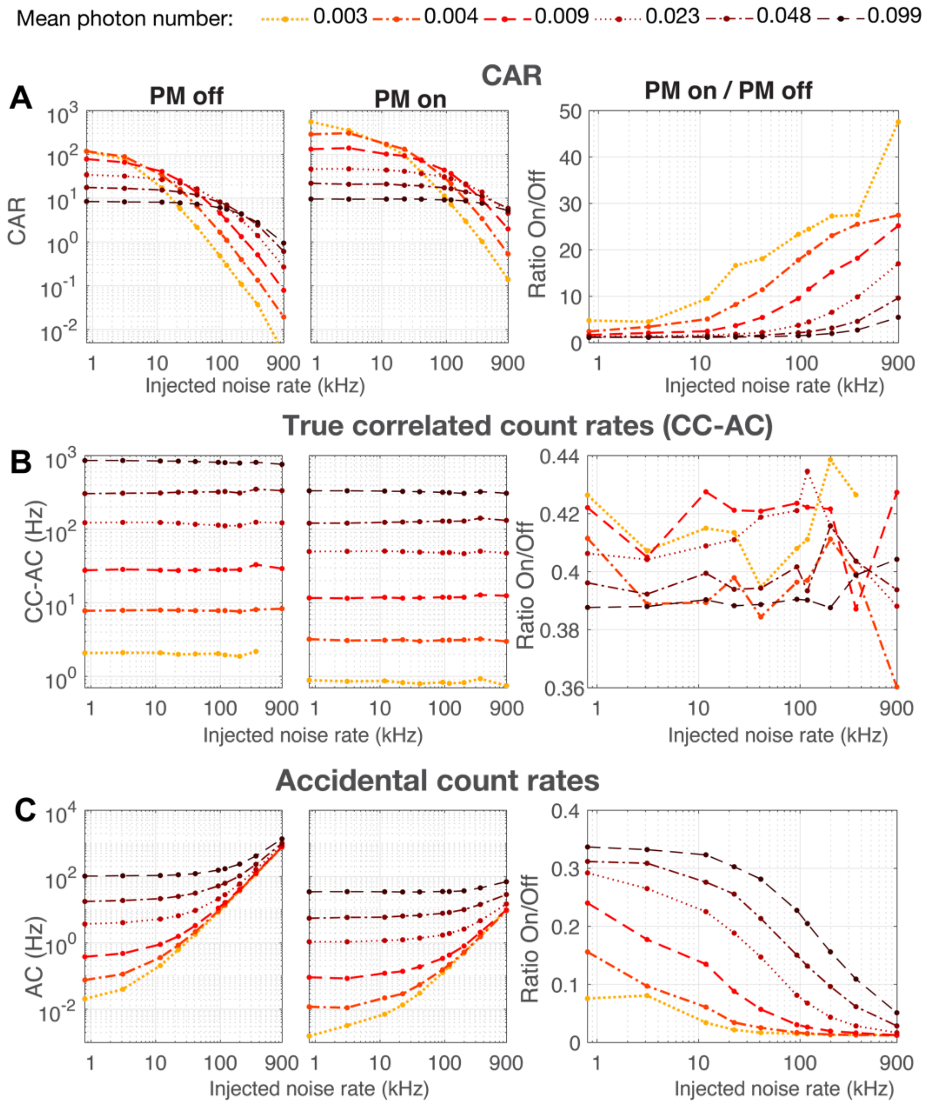

Fig. S13. CAR and related metrics of PM on vs PM off. (**A**) By comparing the output of the TAI with the phase modulation (PM) turned on vs off, the improvement in the energy redistribution effect can be investigated without considering the insertion loss of the system. This leads to a much higher CAR enhancement. (**B**) The reduction of the correlated counts is due to the non-ideal phase modulation, since some of these counts are transferred to the satellite pulses between the TAI peaks formed due to imperfect energy redistribution (see Fig. S1). (**C**) Accidental count rates decrease significantly when the PM is on, leading to the observed CAR enhancements.

6. CAR enhancement vs broadband noise

To further investigate the achieved CAR enhancements depending on noise bandwidth, we carry out a complete analysis of the CAR as a function of mean photon numbers and injected noise rates, where the noise has a bandwidth of 62 GHz, shown in Fig. S14 (comparing PM on vs PM off). The key observation is that very large CAR enhancements can be obtained in this case. In particular, the CAR values for the PM on are higher than the source results with a noise bandwidth of 9-GHz (first column of Fig. 4G of the main text), showing that the qTAI notably outperforms a narrowband filter. For instance, consider the pump setting such that the biphoton has a mean photon number of 0.009, looking at the fourth data point of Fig. 4G of the main text having an injected noise rate of 22.4 kHz. There, we have shown that the output of the qTAI improves the CAR from 47.0 to 92.5 when the noise bandwidth is restricted to 9 GHz. Now, considering the

same mean photon number in Fig. S14 and again the fourth data point, corresponding to the same attenuation on the noise but having a 62 GHz noise bandwidth (i.e., without a narrowband filter) the CAR is still improved from 47.0 to 80.8 despite having a higher injected noise rate of 131.9 kHz. A sample of the histograms is presented in Figs. S15 and S16 for PM off and PM on, respectively. At the considered 62-GHz noise bandwidth, a large portion of the noise is focused in between the TAI peaks, such that it can be very efficiently discriminated from the biphoton through postselection.

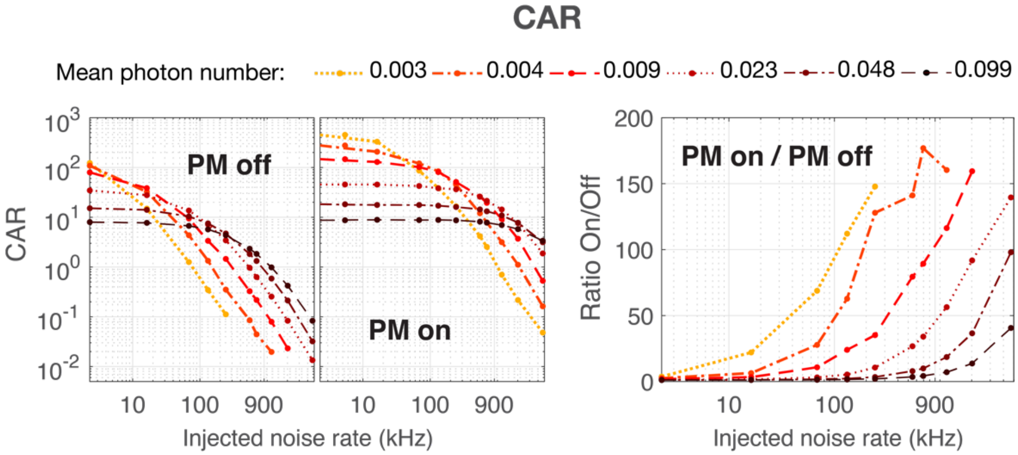

Fig. S14. CAR of PM off vs PM on with a noise bandwidth of 62 GHz. CAR enhancement of 129, from 0.35 to 45.02, obtained at a mean photon number of 0.004 and an injected noise rate of 242.5 kHz. We note that the PM on case has better CAR than the source case with a 9-GHz noise bandwidth (Fig. 4G of the main text), confirming that the SNR improvement from the qTAI outperforms a narrowband filter, even when neglecting the insertion losses from the filter and accounting for those from the qTAI module.

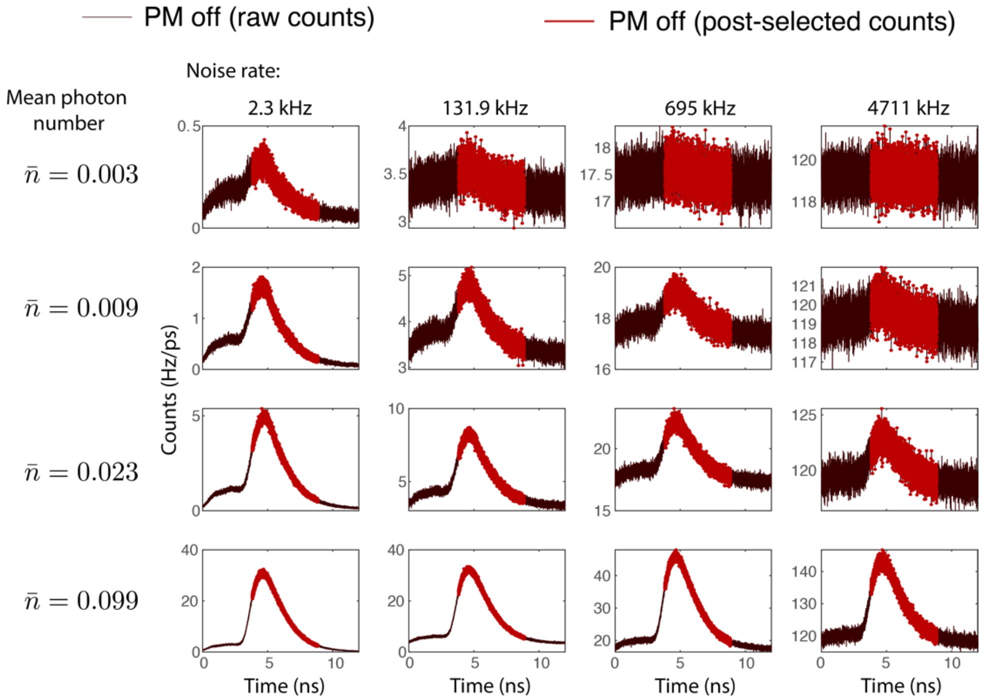

Fig. S15. Sample histograms of the signal photon at the output of the qTAI module, with the phase modulation (PM) turned off. Mean photon numbers are shown increasing from top to bottom, and injected noise rates increasing from left to right. These results correspond to from the experiments reported in the main text, with a noise bandwidth of 62 GHz.

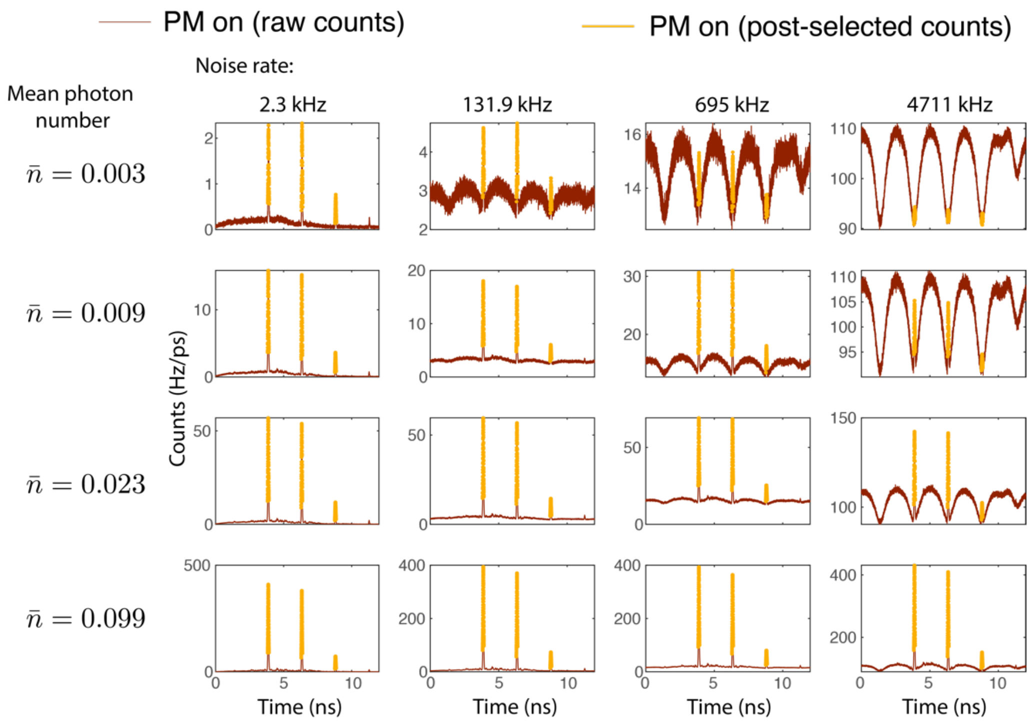

Fig. S16. Sample histograms of signal photon at the output of the qTAI module, with the phase modulation (PM) turned on. Mean photon numbers are shown increasing from top to bottom, and injected noise rates increasing from left to right. These results correspond to from the experiments reported in the main text, with a noise bandwidth of 62 GHz.

REFERENCES

1. J. F. Clauser, M. A. Horne, A. Shimony, R. A. Holt, Proposed experiment to test local hidden-variable theories. *Phys. Rev. Lett.* **23**, 880–884 (1969).

2. T. W. Marshall, E. Santos, F. Selleri, Local realism has not been refuted by atomic cascade experiments. *Phys. Lett. A* **98**, 5–9 (1983).

3. A. Aspect, P. Grangier, About resonant scattering and other hypothetical effects in the orsay atomic-cascade experiment tests of Bell inequalities: A discussion and some new experimental data. *Lett. Nuovo Cimento* **43**, 345–348 (1985).

4. A. Aspect, P. Grangier, G. Roger, Experimental tests of realistic local theories via Bell's theorem. *Phys. Rev. Lett.* **47**, 460–463 (1981).

5. F. Vedovato, C. Agnesi, M. Tomasin, M. Avesani, J.-Å. Larsson, G. Vallone, P. Villoresi, Postselection-loophole-free Bell violation with genuine time-bin entanglement. *Phys. Rev. Lett.* **121**, 190401 (2018).

6. A. Khodadad Kashi, L. Caspani, M. Kues, Spectral Hong-Ou-mandel effect between a heralded single-photon state and a thermal field: Multiphoton contamination and the nonclassicality threshold. *Phys. Rev. Lett.* **131**, 233601 (2023).

7. W. K. Wootters, W. H. Zurek, A single quantum cannot be cloned. *Nature* **299**, 802–803 (1982).

8. S. Wehner, D. Elkouss, R. Hanson, Quantum internet: A vision for the road ahead. *Science* **362**, eaam9288 (2018).

9. S.-K. Liao, W.-Q. Cai, W.-Y. Liu, L. Zhang, Y. Li, J.-G. Ren, J. Yin, Q. Shen, Y. Cao, Z.-P. Li, F.-Z. Li, X.-W. Chen, L.-H. Sun, J.-J. Jia, J.-C. Wu, X.-J. Jiang, J.-F. Wang, Y.-M. Huang, Q. Wang, Y.-L. Zhou, L. Deng, T. Xi, L. Ma, T. Hu, Q. Zhang, Y.-A. Chen, N.-L. Liu, X.-B. Wang, Z.-C. Zhu, C.-Y. Lu, R. Shu, C.-Z. Peng, J.-Y. Wang, J.-W. Pan, Satellite-to-ground quantum key distribution. *Nature* **549**, 43–47 (2017).

10. J. Brendel, N. Gisin, W. Tittel, H. Zbinden, Pulsed energy-time entangled twin-photon source for quantum communication. *Phys. Rev. Lett.* **82**, 2594–2597 (1999).

11. P. Kómár, E. M. Kessler, M. Bishof, L. Jiang, A. S. Sørensen, J. Ye, M. D. Lukin, A quantum network of clocks. *Nat. Phys.* **10**, 582–587 (2014).

12. H.-S. Zhong, H. Wang, Y.-H. Deng, M.-C. Chen, L.-C. Peng, Y.-H. Luo, J. Qin, D. Wu, X. Ding, Y. Hu, P. Hu, X.-Y. Yang, W.-J. Zhang, H. Li, Y. Li, X. Jiang, L. Gan, G. Yang, L. You, Z. Wang, L. Li, N.-L. Liu, C.-Y. Lu, J.-W. Pan, Quantum computational advantage using photons. *Science* **370**, 1460–1463 (2020).

13. P. Kok, W. J. Munro, K. Nemoto, T. C. Ralph, J. P. Dowling, G. J. Milburn, Linear optical quantum computing with photonic qubits. *Rev. Mod. Phys.* **79**, 135–174 (2007).

14. R. Cramer, I. B. Damgrd, J. B. Nielsen, *Secure Multiparty Computation and Secret Sharing* (Cambridge Univ. Press, 2015).

15. S. Du, Y. Tian, Y. Li, Impact of four-wave-mixing noise from dense wavelength-division-multiplexing systems on entangled-state continuous-variable quantum key distribution. *Phys. Rev. Appl.* **14**, 024013 (2020).

16. R. Lin, A. Udalcovs, O. Ozolins, X. Pang, L. Gan, M. Tang, S. Fu, S. Popov, T. F. Da Silva, G. B. Xavier, J. Chen, Telecommunication compatibility evaluation for co-existing quantum key distribution in homogenous multicore fiber. *IEEE Access* **8**, 78836–78846 (2020).

17. L.-J. Wang, K.-H. Zou, W. Sun, Y. Mao, Y.-X. Zhu, H.-L. Yin, Q. Chen, Y. Zhao, F. Zhang, T.-Y. Chen, J.-W. Pan, Long-distance copropagation of quantum key distribution and terabit classical optical data channels. *Phys. Rev. A* **95**, 012301 (2017).

18. R. C. Berrevoets, T. Middelburg, R. F. L. Vermeulen, L. D. Chiesa, F. Broggi, S. Piciaccia, R. Pluis, P. Umesh, J. F. Marques, W. Tittel, J. A. Slater, Deployed measurement-device independent quantum key distribution and Bell-state measurements coexisting with standard internet data and networking equipment. *Commun. Phys.* **5**, 186 (2022).

19. A. Kržič, S. Sharma, C. Spiess, U. Chandrashekara, S. Töpfer, G. Sauer, L. J. González-Martín Del Campo, T. Kopf, S. Petscharnig, T. Grafenauer, R. Lieger, B. Ömer, C. Pacher, R. Berlich, T. Peschel, C. Damm, S. Risse, M. Goy, D. Rieländer, A. Tünnermann, F. Steinlechner, Towards metropolitan free-space quantum networks. *npj Quantum Inf.* **9**, 95 (2023).

20. S. Lloyd, Enhanced sensitivity of photodetection via quantum illumination. *Science* **321**, 1463–1465 (2008).

21. P. S. Blakey, H. Liu, G. Papangelakis, Y. Zhang, Z. M. Léger, M. Lon Iu, A. S. Helmy, Quantum and non-local effects offer over 40 dB noise resilience advantage towards quantum lidar. *Nat. Commun.* **13**, 5633 (2022).

22. D. A. Kalashnikov, A. V. Paterova, S. P. Kulik, L. A. Krivitsky, Infrared spectroscopy with visible light. *Nat. Photonics* **10**, 98–101 (2016).

23. D. C. Lünemann, A. R. Thomas, J. Xu, R. Bartölke, H. Mouritsen, A. De Sio, C. Lienau, Distinguishing between coherent and incoherent signals in excitation-emission spectroscopy. *Opt. Express* **29**, 24326–24337 (2021).

24. H. Xiong, N. Qian, Y. Miao, Z. Zhao, C. Chen, W. Min, Super-resolution vibrational microscopy by stimulated Raman excited fluorescence. *Light Sci. Appl.* **10**, 87 (2021).

25. M. A. Taylor, W. P. Bowen, Quantum metrology and its application in biology. *Phys. Rep.* **615**, 1–59 (2016).

26. M. T. Gruneisen, B. A. Sickmiller, M. B. Flanagan, J. P. Black, K. E. Stoltenberg, A. W. Duchane, Adaptive spatial filtering of daytime sky noise in a satellite quantum key distribution downlink receiver. *Opt. Eng.* **55**, 026104 (2016).

27. F. Bouchard, D. England, P. J. Bustard, K. L. Fenwick, E. Karimi, K. Heshami, B. Sussman, Achieving ultimate noise tolerance in quantum communication. *Phys. Rev. Appl.* **15**, 024027 (2021).

28. J.-H. Kim, Y. Kim, D.-G. Im, C.-H. Lee, J.-W. Chae, G. Scarcelli, Y.-H. Kim, Noise-resistant quantum communications using hyperentanglement. *Optica* **8**, 1524–1531 (2021).

29. C. Reimer, S. Sciara, P. Roztocki, M. Islam, L. Romero Cortés, Y. Zhang, B. Fischer, S. Loranger, R. Kashyap, A. Cino, S. T. Chu, B. E. Little, D. J. Moss, L. Caspani, W. J. Munro, J. Azaña, M. Kues, R. Morandotti, High-dimensional one-way quantum processing implemented on *d*-level cluster states. *Nat. Phys.* **15**, 148–153 (2018).

30. T. Dai, Y. Ao, J. Bao, J. Mao, Y. Chi, Z. Fu, Y. You, X. Chen, C. Zhai, B. Tang, Y. Yang, Z. Li, L. Yuan, F. Gao, X. Lin, M. G. Thompson, J. L. O'Brien, Y. Li, X. Hu, Q. Gong, J. Wang, Topologically protected quantum entanglement emitters. *Nat. Photonics* **16**, 248–257 (2022).

31. A. W. Lohmann, J. A. Thomas, Making an array illuminator based on the Talbot effect. *Appl. Opt.* **29**, 4337–4340 (1990).

32. J. Nuño, C. Finot, J. Fatome, Linear sampling and magnification technique based on phase modulators and dispersive elements: The temporal lenticular lens. *Opt. Fiber Technol.* **36**, 125–129 (2017).

33. B. H. Kolner, Space-time duality and the theory of temporal imaging. *IEEE J. Sel. Top. Quantum Electron.* **30**, 1951–1963 (1994).

34. J. M. Lukens, D. E. Leaird, A. M. Weiner, A temporal cloak at telecommunication data rate. *Nature* **498**, 205–208 (2013).

35. L. Romero Cortés, M. Seghilani, R. Maram, J. Azaña, Full-field broadband invisibility through reversible wave frequency-spectrum control. *Optica* **5**, 779–786 (2018).

36. B. Crockett, L. Romero Cortés, S. R. Konatham, J. Azaña, Full recovery of ultrafast waveforms lost under noise. *Nat. Commun.* **12**, 2402 (2021).

37. B. Crockett, L. Romero Cortés, R. Maram, J. Azaña, Optical signal denoising through temporal passive amplification. *Optica* **9**, 130–138 (2022).

38. J. Azaña, X. Zhu, C. Rowe, B. Crockett, Optical time-mapped spectrograms (II): fractional Talbot designs. *J. Lightwave Technol.* **41**, 5284–5295 (2023).

39. C. M. L. Rowe, B. Crockett, X. Zhu, J. Azaña, Versatile photonic spectrograms for ultrafast real-time broadband microwave signal analysis. *IEEE Trans. Microw. Theory Tech.* **73**, 3424–3441 (2025).

40. J. M. Lukens, O. Odele, C. Langrock, M. M. Fejer, D. E. Leaird, A. M. Weiner, Generation of biphoton correlation trains through spectral filtering. *Opt. Express* **22**, 9585–9596 (2014).

41. M. Karpiński, M. Jachura, L. J. Wright, B. J. Smith, Bandwidth manipulation of quantum light by an electro-optic time lens. *Nat. Photonics* **11**, 53–57 (2017).

42. F. Sośnicki, M. Mikołajczyk, A. Golestani, M. Karpiński, Interface between picosecond and nanosecond quantum light pulses. *Nat. Photonics* **17**, 761–766 (2023).

43. C. Rowe, X. Zhu, B. Crockett, G. Lim, M. Goodarzi, M. Fernández, J. Van Howe, H. Sun, S. Kaushal, A. Shoeib, J. Azaña, Linear optical wave energy redistribution methods for photonic signal processing. *npj Nanophotonics* **2**, 13 (2025).

44. S. Mittal, V. V. Orre, A. Restelli, R. Salem, E. A. Goldschmidt, M. Hafezi, Temporal and spectral manipulations of correlated photons using a time lens. *Phys. Rev. A* **96**, 043807 (2017).

45. L. Romero Cortés, H. Guillet de Chatellus, J. Azaña, On the generality of the Talbot condition for inducing self-imaging effects on periodic objects. *Opt. Lett.* **41**, 340–343 (2016).

46. J. Wen, Y. Zhang, M. Xiao, The Talbot effect: Recent advances in classical optics, nonlinear optics, and quantum optics. *Adv. Opt. Photonics* **5**, 83–130 (2013).

47. A. V. Oppenheim, A. S. Willsky, S. H. Nawab, *Signals & Systems* (Prentice-Hall Inc., 1996).

48. C. R. Fernández-Pousa, R. Maram, J. Azaña, CW-to-pulse conversion using temporal Talbot array illuminators. *Opt. Lett.* **42**, 2427–2430 (2017).

49. O. Kuzucu, F. N. C. Wong, S. Kurimura, S. Tovstonog, Joint temporal density measurements for two-photon state characterization. *Phys. Rev. Lett.* **101**, 153602 (2008).

50. K. Zielnicki, K. Garay-Palmett, D. Cruz-Delgado, H. Cruz-Ramirez, M. F. O'Boyle, B. Fang, V. O. Lorenz, A. B. U'Ren, P. G. Kwiat, Joint spectral characterization of photon-pair sources. *J. Mod. Opt.* **65**, 1141–1160 (2018).

51. M. V. Berry, S. Klein, Integer, fractional and fractal Talbot effects. *J. Mod. Opt.* **43**, 2139–2164 (1996).

52. C. R. Fernández-Pousa, On the structure of quadratic Gauss sums in the Talbot effect. *J. Opt. Soc. Am. A* **34**, 732–742 (2017).

53. H. Yu, B. Crockett, N. Montaut, S. Sciara, M. Chemnitz, S. T. Chu, B. E. Little, D. J. Moss, Z. Wang, J. Azaña, R. Morandotti, Exploiting nonlocal correlations for dispersion-resilient quantum communications. *Phys. Rev. Lett.* **134**, 220801 (2025).

54. M. Kues, C. Reimer, P. Roztocki, L. Romero Cortés, S. Sciara, B. Wetzel, Y. Zhang, A. Cino, S. T. Chu, B. E. Little, D. J. Moss, L. Caspani, J. Azaña, R. Morandotti, On-chip generation of high-dimensional entangled quantum states and their coherent control. *Nature* **546**, 622–626 (2017).

55. J. A. Jaramillo-Villegas, P. Imany, O. D. Odele, D. E. Leaird, Z.-Y. Ou, M. Qi, A. M. Weiner, Persistent energy–time entanglement covering multiple resonances of an on-chip biphoton frequency comb. *Optica* **4**, 655–658 (2017).

56. N. Maring, P. Farrera, K. Kutluer, M. Mazzera, G. Heinze, H. de Riedmatten, Photonic quantum state transfer between a cold atomic gas and a crystal. *Nature* **551**, 485–488 (2017).

57. J. Azaña, Temporal self-imaging effects for periodic optical pulse sequences of finite duration. *J. Opt. Soc. Am. B* **20**, 83–90 (2003).

58. M. A. Muriel, J. Azaña, A. Carballar, Real-time Fourier transformer based on fiber gratings. *Opt. Lett.* **24**, 1–3 (1999).

59. W. C. Jiang, X. Lu, J. Zhang, O. Painter, Q. Lin, Silicon-chip source of bright photon pairs. *Opt. Express* **23**, 20884–20904 (2015).

60. A. M. Weiner, Ultrafast optical pulse shaping: A tutorial review. *Opt. Commun.* **284**, 3669–3692 (2011).

61. E. Meyer-Scott, C. Silberhorn, A. Migdall, Single-photon sources: Approaching the ideal through multiplexing. *Rev. Sci. Instrum.* **91**, 041101 (2020).

62. S. Ecker, F. Bouchard, L. Bulla, F. Brandt, O. Kohout, F. Steinlechner, R. Fickler, M. Malik, Y. Guryanova, R. Ursin, M. Huber, Overcoming noise in entanglement distribution. *Phys. Rev. X* **9**, 041042 (2019).

63. W. Tittel, J. Brendel, H. Zbinden, N. Gisin, Violation of Bell inequalities by photons more than 10 km apart. *Phys. Rev. Lett.* **81**, 3563–3566 (1998).

64. H. De Riedmatten, I. Marcikic, V. Scarani, W. Tittel, H. Zbinden, N. Gisin, Tailoring photonic entanglement in high-dimensional Hilbert spaces. *Phys. Rev. A* **69**, 050304 (2004).

65. Q. Zhang, C. Langrock, H. Takesue, X. Xie, M. Fejer, Y. Yamamoto, Generation of 10-GHz clock sequential time-bin entanglement. *Opt. Express* **16**, 3293–3298 (2008).

66. D. F. V. James, P. G. Kwiat, W. J. Munro, A. G. White, Measurement of qubits. *Phys. Rev. A* **64**, 052312 (2001).

67. H. Takesue, Y. Noguchi, Implementation of quantum state tomography for time-bin entangled photon pairs. *Opt. Express* **17**, 10976–10989 (2009).

68. B. Crockett, J. van Howe, N. Montaut, R. Morandotti, J. Azaña, High-resolution time-correlated single-photon counting using electro-optic sampling. *Laser Photonics Rev.* **16**, 2100635 (2022).

69. M. Yu, D. Barton III, R. Cheng, C. Reimer, P. Kharel, L. He, L. Shao, D. Zhu, Y. Hu, H. R. Grant, L. Johansson, Y. Okawachi, A. L. Gaeta, M. Zhang, M. Lončar, Integrated femtosecond pulse generator on thin-film lithium niobate. *Nature* **612**, 252–258 (2022).

70. M. P. Fernández, S. Kaushal, B. Crockett, L. A. Bulus-Rossini, P. A. Costanzo-Caso, J. Azaña, An ultra-fast temporal Talbot array illuminator. *J. Lightwave Technol.* **41**, 4725–4733 (2023).

71. A. Opremcak, I. V. Pechenezhskiy, C. Howington, B. G. Christensen, M. A. Beck, E. Leonard Jr., J. Suttle, C. Wilen, K. N. Nesterov, G. J. Ribeill, T. Thorbeck, F. Schlenker, M. G. Vavilov, B. L. T. Plourde, R. McDermott, Measurement of a superconducting qubit with a microwave photon counter. *Science* **361**, 1239–1242 (2018).

72. C. D. Bruzewicz, J. Chiaverini, R. McConnell, J. M. Sage, Trapped-ion quantum computing: Progress and challenges. *Appl. Phys. Rev.* **6**, 021314 (2019).

73. T. Ren, M. Zhang, C. Wang, L. Shao, C. Reimer, Y. Zhang, O. King, R. Esman, T. Cullen, M. Loncar, An integrated low-voltage broadband lithium niobate phase modulator. *IEEE Photonics Technol. Lett.* **31**, 889–892 (2019).

74. Gracy-Fiber, "Low loss polarization maintaining optical circulator" (2025); www.gracy-fiber.com/micro-optic/pm-series/polarization-maintaining-optical-circulator.html.

75. J. F. Brennan, M. R. Matthews, W. V. Dower, D. J. Treadwell, W. Wang, J. Porque, X. Fan, Dispersion correction with a robust fiber grating cover the full c-band at 10-gb/s rates with <0.3-dB power penalties. *IEEE Photonics Technol. Lett.* **15**, 1722–1724 (2003).

76. J. Ashby, V. Thiel, M. Allgaier, P. d'Ornellas, A. O. C. Davis, B. J. Smith, Temporal mode transformations by sequential time and frequency phase modulation for applications in quantum information science. *Opt. Express* **28**, 38376–38389 (2020).

77. A. O. C. Davis, V. Thiel, B. J. Smith, Measuring the quantum state of a photon pair entangled in frequency and time. *Optica* **7**, 1317–1322 (2020).

78. T.-H. Chang, B. M. Fields, M. E. Kim, C.-L. Hung, Microring resonators on a suspended membrane circuit for atom–light interactions. *Optica* **6**, 1203–1210 (2019).

79. C. Reimer, L. Caspani, M. Clerici, M. Ferrera, M. Kues, M. Peccianti, A. Pasquazi, L. Razzari, B. E. Little, S. T. Chu, D. J. Moss, R. Morandotti, Integrated frequency comb source of heralded single photons. *Opt. Express* **22**, 6535–6546 (2014).

80. D. N. Klyshko, Use of two-photon light for absolute calibration of photoelectric detectors. *Sov. J. Quantum Electron.* **10**, 1112 (1980).

81. P. Roztocki, B. MacLellan, M. Islam, C. Reimer, B. Fischer, S. Sciara, R. Helsten, Y. Jestin, A. Cino, S. T. Chu, B. Little, D. J. Moss, M. Kues, R. Morandotti, Arbitrary phase access for stable fiber interferometers. *Laser Photonics Rev.* **15**, 2000524 (2021).

82. J.-H. Kim, J.-W. Chae, Y.-C. Jeong, Y.-H. Kim, Quantum communication with time-bin entanglement over a wavelength-multiplexed fiber network. *APL Photonics* **7**, 016106 (2022).

83. B. Crockett, C. Rowe, J. Azaña, Capturing ultra-broadband complex-fields of arbitrary duration using a real-time spectrogram. *APL Photonics* **8**, 066108 (2023).

84. J. Azaña, M. A. Muriel, Temporal self-imaging effects: Theory and application for multiplying pulse repetition rates. *IEEE J. Sel. Top. Quantum Electron.* **7**, 728–744 (2001).

85. W. Wen, Z. Chen, L. Lu, W. Yan, W. Xue, P. Zhang, Y. Lu, S. Zhu, X. Ma, Realizing an entanglement-based multi-user quantum network with integrated photonics. *Phys. Rev. Appl.* **18**, 024059 (2022).

86. T. J. Steiner, M. Shen, J. E. Castro, J. E. Bowers, G. Moody, Continuous entanglement distribution from an AlGaAs-on-insulator microcomb for quantum communications. *Opt. Quantum* **1**, 55–62 (2023).

87. C. Erven, X. Ma, R. Laflamme, G. Weihs, Entangled quantum key distribution with a biased basis choice. *New J. Phys.* **11**, 045025 (2009).

88. H. Takesue, K. Inoue, 1.5-μm Band quantum-correlated photon pair generation in dispersion-shifted fiber: Suppression of noise photons by cooling fiber. *Opt. Express* **13**, 7832–7839 (2005).
